# Atopic dermatitis and risk of autoimmune diseases: a systematic review and meta-analysis

**DOI:** 10.3389/fimmu.2025.1539997

**Published:** 2025-06-12

**Authors:** Hongli Wang, Min Chen, Tengyue Wang, Wenyu Cai, Xuanlin Li, Lin Huang, Mingzhu Wang

**Affiliations:** ^1^ Innovative Research Center for Basic Medicine on Autoimmune Diseases, Ministry of Education, Hangzhou, Zhejiang, China; ^2^ Key Laboratory of Chinese Medicine Rheumatology of Zhejiang Province, College of Basic Medical Science, Zhejiang Chinese Medical University, Hangzhou, Zhejiang, China; ^3^ Department of Party and Government Comprehensive Office, The First Affiliated Hospital of Zhejiang Chinese Medical University, Hangzhou, Zhejiang, China; ^4^ School of Pharmaceutical Sciences, Zhejiang Chinese Medical University, Hangzhou, China

**Keywords:** atopic dermatitis, autoimmune diseases, meta-analysis, adults, children cohort studies (n=19)

## Abstract

**Background:**

Atopic dermatitis (AD) is a common recurrent chronic inflammatory skin disease, and there is increasing evidence of a possible association between AD and autoimmune diseases.

**Objectives:**

This study aimed to summarize existing epidemiological studies on the association between AD and autoimmune diseases and to perform a meta-analysis of combinable results.

**Methods:**

We conducted a thorough search for cohort studies, case-control studies and cross-sectional studies across the PubMed, Cochrane Library, and Embase databases, from their inception to May 24, 2024, using medical subject headings and relevant keywords. All data were meticulously analyzed using Stata statistical software version 17.0. The protocol was registered on PROSPERO (CRD42024547282).

**Result:**

A total of 26 cohort studies, comprising 1,629,723 patients with atopic dermatitis and 15,106,889 control subjects, were included in this meta-analysis. These studies were published between 2014 and 2024 and included 19 cohort studies, 2 case-control studies, and 5 cross-sectional studies. The current study demonstrated a significant association of atopic dermatitis with autoimmune diseases[HR 1.49, 95% CI (1.31-1.70); *P<*0.001], including celiac disease, systemic lupus erythematosus, Sjogren’s syndrome, ankylosing spondylitis, alopecia areata, rheumatoid arthritis, vitiligo, thyroid dysfunction, ulcerative colitis.

**Conclusion:**

The results of our study indicate a clear association between atopic dermatitis and autoimmune diseases, both in adults and children. Additionally, women were more likely to have autoimmune disease complications than men. However, due to the limited number of participants in our study, further research is needed to thoroughly investigate the relationship.

**Systematic review registration:**

https://www.crd.york.ac.uk/PROSPERO/, identifier CRD42024547282.

## Introduction

Atopic dermatitis (AD) is a recurrent, chronic inflammatory skin disease that affects approximately 20% of children and 7 to 10% of adults in high-income countries. It is also prevalent in developing world, posing a significant public health concern due to its presence and increasing prevalence across most countries (1, 2). The defining features of AD include generalized dry skin, recurrent eczematous lesions, and pruritus (3). These symptoms can significantly impact daily activities, potentially leading to sleep disorders, thereby reducing an individual’s quality of life (4). The severity of the disease correlates with the frequency of recurrences and healthcare utilization, imposing a substantial financial burden on patients (5).

Evidence suggests that AD may possess an autoimmune component, with disease progression resembling that of known autoimmune disorders characterized by alternating relapse and remission phases. Furthermore, significant associations have been identified between AD and multiple autoimmune diseases (6). Autoimmune diseases represent a group of chronic, systemic disorders characterized by aberrant immune responses, excessive inflammation, and widespread deposition of immune complexes in tissues and organs. Epidemiological studies indicate that these conditions affect approximately 5–8% of the global population, highlighting their significant public health burden (7). A Mendelian randomization analysis has demonstrated that atopic dermatitis (AD) significantly increases the risk of rheumatoid arthritis (RA), type 1 diabetes (T1D), and autoimmune alopecia (AA), supporting a substantial causal relationship between these conditions. Although the precise pathogenic mechanisms linking AD to RA, T1D, and AA remain unclear, emerging evidence suggests that immune dysregulation and shared genetic susceptibility may underlie this association (8).

Several large-scale population-based studies have recently reported associations between AD and multiple autoimmune diseases. A meta-analysis on a similar topic was published in 2021 (9), however, the subgroup analyses did not stratify by age, sex, and AD severity. Simultaneously, numerous well-executed cohort studies have recently been published, presenting new evidence regarding the association between AD and autoimmune diseases. Given the importance of this subject, the limitations of prior reviews, and the availability of new data, we conducted a systematic review and meta-analysis to evaluate the association between AD and autoimmune diseases in adults compared to children.

## Materials and methods

This meta-analysis was conducted in accordance with the Preferred Reporting Items for Systematic Reviews and Meta-Analyses (PRISMA) 2020 guidelines (10).

### Data sources

A thorough search encompassing the PubMed, Cochrane Library and Embase databases was conducted for cohort studies from the database’s inception to 26 May 2024, without any restrictions applied. Subject terms (Embase: Emtree; PubMed: MeSH) and keywords were utilized to identify relevant studies. The search terms comprised terms associated with atopic dermatitis, chronic atopy, autoimmune diseases, immune system disorders, autoantibodies, autoimmunity and specific autoimmune diseases. The detailed search strategy for the three databases is outlined in **Supplementary Tables 1**-**3**.

### Eligibility criteria

The studies included in this analysis fulfilled the following eligibility criteria: (1) Population: This study includes individuals of all ages, both children and adults. The diagnosis of ADwas based on ICD-10-CM or ICD-9-CM codes.(2) Exposure: Diagnosis of AD. (3) Comparator: Individuals without AD (general population or healthy controls). (4) Outcomes: Incidence of autoimmune diseases in AD patients compared to the non-AD population. (5)Study Design: Eligible studies include cohort studies, case-control studies, and cross-sectional studies. (6) Exclusion criteria: conference abstracts; duplicate publications; incomplete data or no results of interest.

### Study selection

Two reviewers, HL and WMZ, were responsible for independently screening and selecting eligible records based on the established eligibility criteria. The initial screening involved excluding duplicate records and irrelevant articles based on the titles and abstracts. In the second stage, the full-text articles were downloaded and reviewed to determine which studies could be included in the meta-analysis. Any discrepancies between the two reviewers during the study selection process were resolved through group discussion.

### Data extraction

A data extraction form was designed in Microsoft Excel by three authors, WHL, CM, WTY and CWY. The principal elements extracted were the first author, publication date, country, age, and diagnosis of atopic dermatitis. The extracted data were subjected to a rigorous cross-checking process, with any discrepancies resolved through discussion.

### Quality assessment and risk of bias

**Figure 1:**
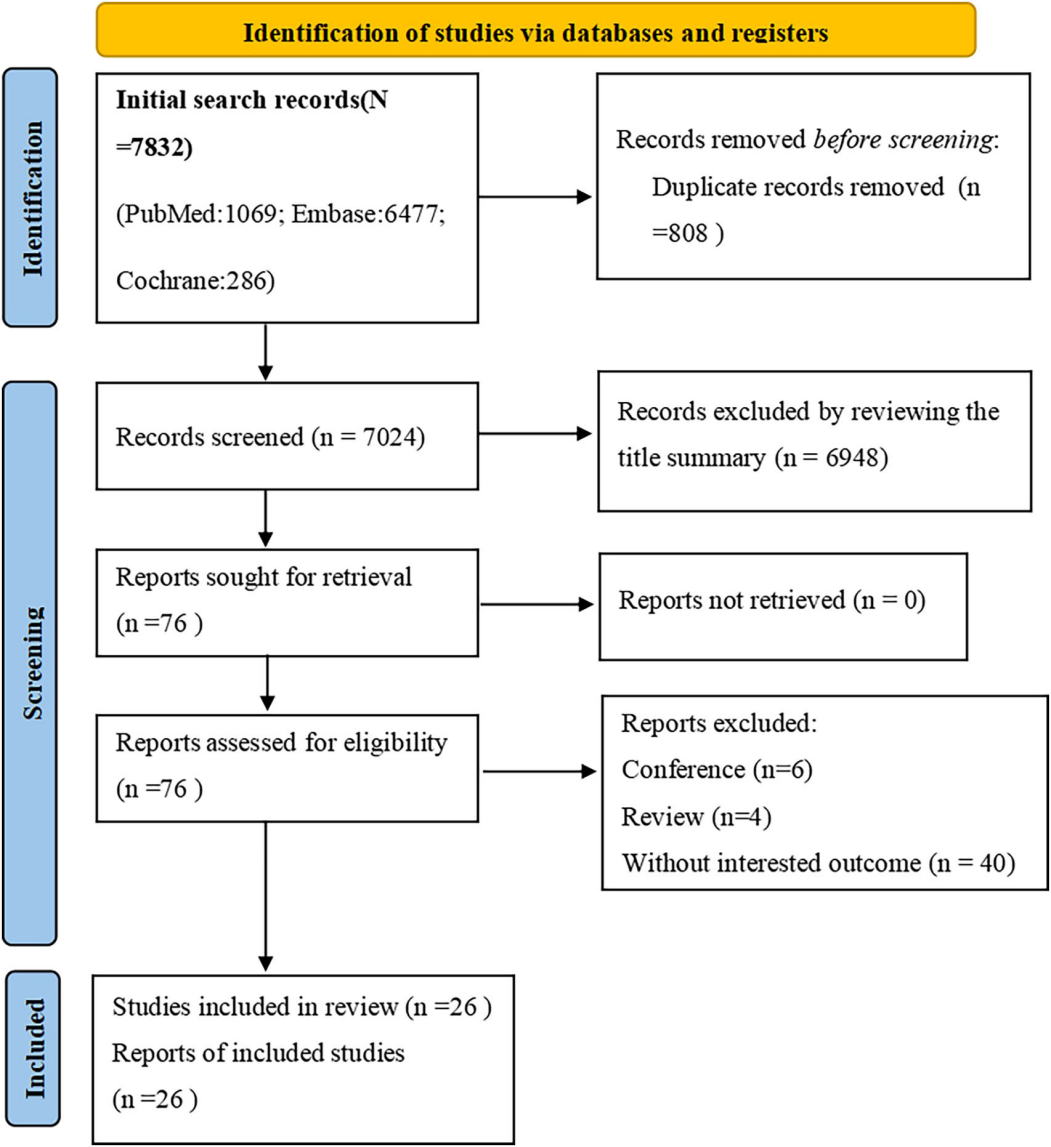
Studies screening process. PRISMA flow diagram of the screening and selection process according to PRISMA 2020 guidelines.

Assessment: The quality of the included cohort studies, case-control studies and cross-sectional studies were evaluated using the Newcastle-Ottawa Scale (NOS) (11). The NOS employs a star-based system, with a maximum score of 9 stars. The stars are awarded based on the following criteria: Selection (4 stars): Represents the quality of participant selection and measurement of exposure; Comparability (2 stars): Reflects the comparability of the study design and statistical analyses; Outcome (3 stars): Evaluates the adequacy of the outcome indicator and the length of follow-up. The number of stars assigned to each study corresponds to the quality of the study. Studies with 0–3 stars were considered to be of low quality, those with 4–6 stars were deemed to be of moderate quality, and high-quality studies were those with 7–9 stars. The results of the quality assessment are provided in **Table 1** (9). Additionally, PRISMA tables (**Figure 1**) were constructed to report the meta-analysis in a standardized format.

**Table 1:** Characteristics of studies.

Author	Year	Country	Study type	Sample size		Age
				AD	control	
Jungho Ahn (12)	2024	Korea	A national administrative cohort study	39,832	159,328	≤18 years
Ju Hee Kim	2023	Korea	A national administrative cohort study	67,632	270,528	≤18 years
Brandon Smith (14)	2023	the United States	Retrospective cohort study	1,060	9,000	20–59 years
Tejas P (15)	2023	the United States	A nested case-control study	13,756	55,024	47.2 ± 18.2years
Paula L Keskitalo (16)	2023	Finland	Retrospective cohort study	70,584	270,783	≤18 years
Meng-Chieh Li	2023	China	Retrospective cohort study	396,461	1,585,844	3.8 ± 4.7 years
Simon de Lusignan (18)	2022	the United Kingdom	Retrospective cohort analysis	173,709	694,836	27.6 ± 28.6 years
Saman Mohammadi	2022	Iran	A cross-sectional study	62	62	12–18 years
Youkyung S (20)	2022	the United States	Retrospective cohort analysis	397,79	353,743	18–64 years
Ying-Xiu Dai (21)	2021	China	A Nationwide Population-Based Cohort Study	8,206	32,824	42.4 (29.2–56.1) years
Amy H	2021	the United States	Retrospective cohort analysis	86,969	116,564	≤18 years
L.U. Ivert (23)	2021	Swedish	Population-based case–control study	104,832	1,022,435	34.9 (17.8) years
Hosim Soh (24)	2021	Korea	Retrospective cohort study	40,777	9,882,744	≥20 years
Saana Kauppi (25)	2021	Finland	Retrospective cohort study	94,975	228,642	≤18 years
Yu-Hsun Wei (26)	2020	China	Bidirectional cohort study	240,307	161,228	30.8 (22.5-41.2) years
Guy Shalom	2019	Israel	Cross-sectional observational study	116,816	116,812	>18 years
Kauppi, S (28)	2019	Finland	Retrospective cohort study	96,066	250,000	≤18 years
Treudler R (29)	2018	Germany	Cross-sectionally study	372	9,109	40–79 years
Shanthi Narla	2018	the United States	Cross-sectional study	9,290	44,605	All ages
Jonathan I. Silverberg (31)	2018	the United States	Cross-sectional US population-based study	496	1,974	Average age 52 years
Alexander Egeberg (32)	2017	Denmark	Retrospective cohort study	7,937	79,370	≥18 years
Chang-Ching Wei (33)	2016	China	Retrospective cohort study	120,704	241,408	4.95 ± 4.85 years
Yuki M. F. Andersen	2016	Denmark	Retrospective cohort study	8,112	40,560	42.42 (15.16) years
Chien-Heng Lin (35)	2016	China	Retrospective cohort study	90	1,555	≤18 years
Jochen Schmitt	2015	Germany	Retrospective cohort study	49,847	605,968	≤40 years
Lung-Chi Wu (37)	2014	China	Retrospective cohort study	41,950	167,800	34.71 (23.79) years

A subgroup analysis was conducted according to the following criteria: specific autoimmune disease, age, sex, and study design. In the subgroup analysis of age, the risk of onset of autoimmune diseases in adults and children is initially examined. Subsequently, the risk of the onset of specific autoimmune diseases in adults and children is analyzed.

### Statistical analysis

All statistical analyses were conducted using Stata Statistical Software, version 17.0. The adjusted Hazard Ratio (HR) and its corresponding 95% confidence interval (CI) were employed to evaluate the correlation between atopic dermatitis and the risk of autoimmune disease. To assess heterogeneity, the *I²* statistic was employed. In consideration of the degree of heterogeneity identified, a random effects model was selected for analysis in instances where *I²* exceeded 50%, while a fixed effects model was employed when *I²* was below this threshold.

A sensitivity analysis was conducted to ensure the robustness of the overall results. The potential for publication bias was evaluated through a visual examination of the funnel plot and a statistical assessment using Egger regression.

## Results

### Literature search

The systematic search for studies published prior to May 26, 2024 yielded a total of 7,832 results. After the initial screening, 808 duplicate records were excluded. Additionally, 6872 articles were removed based on the screening of titles and abstracts, as they were deemed unrelated to the topic. The remaining 76 studies underwent a full-text review. Finally, 26 cohort studies were identified that reported on the association between atopic dermatitis and the risk of autoimmune disease flare-ups. The detailed study selection process is illustrated in **Figure 1** (12–37).

### Study characteristics

This meta-analysis included a total of 26 studies published between 2014 and 2024, comprising 19 cohort studies, 2 case-control studies, and 5 cross-sectional studies. The age distribution of the included populations was as follows: 9 studies with populations younger than 18 years of age, 8 studies with populations older than 18 years of age, 8 studies included participants across all age groups. In terms of the geographic distribution, the studies were conducted in the following countries: 6 studies each from China and the United States, 3 studies each from Korea and Finland, 2 studies each from Germany and Denmark, 1 study each from the United Kingdom, Israel, Switzerland, and Iran. All the included studies provided adjusted estimates, although the specific confounding factors adjusted for (e.g., age, sex, marital status, education level) varied slightly across the studies. The main characteristics of the included studies are shown in **Table 1**. Due to space limitations, additional details will be provided in **Supplementary Table 4**.

### Quality assessment

The mean score for all included cohort studies was 7.69 based on the NOS criteria. In excess of 88% of studies achieved a score of 6 or above, with over 50% scoring 7 or above. The included scores are presented in **Table 2**.

**Table 2:** The Newcastle-Ottawa scale.

Study	Year	Selection	Comparability	Outcome	Total
Cohort studies (n=19)					
Jungho Ahn (12)	2024	***	**	***	8
Ju Hee Kim	2023	***	**	***	8
Meng-Chieh Li	2023	**	**	**	6
Brandon Smith (14)	2023	**	**	**	6
Paula L Keskitalo (16)	2023	**	**	**	6
Simon de Lusignan (18)	2022	***	**	***	8
Youkyung S (20)	2022	***	**	***	8
Ying-Xiu Dai (21)	2021	**	**	**	6
Amy H	2021	***	**	***	8
Hosim Soh (24)	2021	**	**	**	6
Saana Kauppi (25)	2021	**	**	*	5
Yu-Hsun Wei (26)	2020	**	*	**	5
Kauppi, S (28)	2019	**	**	**	6
Alexander Egeberg (32)	2017	**	**	***	7
Yuki M. F. Andersen	2016	***	**	***	8
Chang-Ching Wei (33)	2016	**	*	**	5
Chien-Heng Lin (35)	2016	**	**	**	6
Jochen Schmitt	2015	**	**	***	7
Lung-Chi Wu (37)	2014	**	**	***	7
Case-control studies (n=2)					
Tejas P (15)	2023	***	**	**	7
L.U. Ivert (23)	2021	***	***	***	8
Cross-sectional studies (n=5)					
Saman Mohammadi	2022	**	**	**	6
Guy Shalom	2019	**	**	**	6
Treudler R (29)	2018	**	**	**	6
Shanthi Narla	2018	***	**	***	8
Jonathan I. Silverberg (31)	2018	***	**	**	7

The stars are the symbols commonly used in the evaluation of NOS scale.

### Atopic dermatitis and risk of autoimmune disease

**Figure 2:**
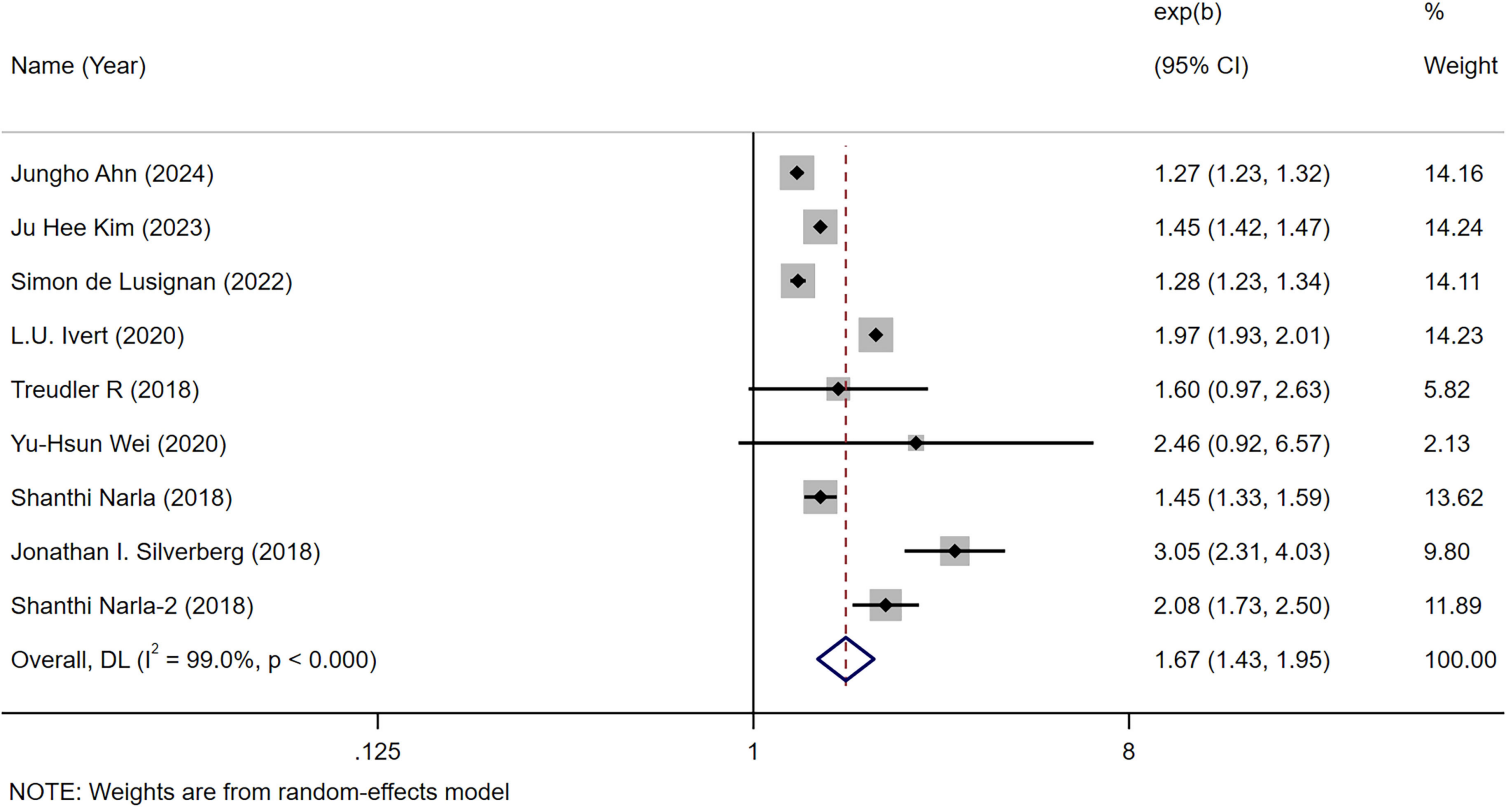
Meta-analysis of the risk of autoimmune disease associated with atopic dermatitis.

We hereby clarify that among the 26 studies included in the meta-analysis, only 9 studies explicitly reported HRs quantifying the association between AD and autoimmune diseases (collective assessment rather than disease-specific evaluation). The remaining studies primarily examined associations between AD and specific autoimmune diseases (e.g., Crohn’s disease, alopecia areata, etc.). Consequently, we conducted in-depth secondary analyses focusing specifically on these 9 studies. The combined analyses demonstrated a significant correlation between atopic dermatitis and an increased risk of autoimmune disease [OR = 1.67, 95% CI (1.43-1.95), *I²* = 99.0%, *P<*0.001] (12–37) (**Figure 2**). One of the articles included the risk of both AD and autoimmune disease in children and adults. This was done because the association between AD and autoimmune disease was analyzed across all ages.

### Atopic dermatitis and risk of specific autoimmune disease

The meta-analysis identified a total of 9 studies that investigated the association between atopic dermatitis and the following autoimmune conditions: coeliac disease (9 studies) (20, 22, 23, 25, 27, 28, 30, 34), Crohn’s disease (9 studies) (12, 18, 23, 24, 30, 32, 34, 36),ulcerative colitis (9 studies) (12, 18, 23, 24, 30, 32, 34, 36), alopecia areata (8 studies) (18, 20, 22, 23, 26, 30, 34), thyroid dysfunction (8 studies) (12, 13, 18, 23, 26, 29–31), rheumatoid arthritis (7 studies) (23, 29, 30, 34, 36, 37), psoriasis (6 studies) (12, 13, 21–23, 26), systemic lupus erythematosus (6 studies) (12, 20, 23, 30, 34), idiopathic thrombocytopenic purpura (5 studies) (12, 13, 30, 33, 34), Sjogren’s syndrome (4 studies) (12, 20, 30, 34) and ankylosing spondylitis (4 studies) (12, 23, 30, 34).

The combined analysis revealed that AD was most strongly associated with an elevated risk of alopecia areata [HR = 7.80, 95% CI (4.39-13.86), *I²* = 97.80%, *P<*0.001]. The results demonstrated that AD was significantly associated with an increased risk of psoriasis [HR = 3.39, 95% CI (2.17-5.31), *I²* = 97.20%, *P<*0.001], celiac disease [HR = 2.12, 95% CI (1.88-2.38), *I²* = 80.9%, *P<*0.001], vitiligo [HR = 2.34, 95% CI (1.72-3.19), *I²* = 95.70%, *P<*0.001]. Furthermore, AD was also found to be significantly associated with an increased risk of systemic lupus erythematosus [HR=1.87,95%CI(1.48-2.35,*I²* = 80.2%, *P<*0.001], Sjogren’s syndrome [HR = 2.08, 95% CI (1.45-3.00), *I²* = 77.50%, *P<*0.001], ankylosing spondylitis [HR = 1.65, 95% CI (1.30-2.09), *I²* = 48.60%, *P<*0.001], rheumatoid arthritis (HR = 1.47, 95% CI (1.39-1.56), *I²* = 0.00%, *P<*0.001], ankylosing spondylitis [HR = 1.65, 95% CI (1.30-2.10), *I²* = 48.60%, *P<*0.001], Crohn’s disease [HR= 1.50, 95% CI (1.23-1.83), *I²* = 85.70%, *P<*0.001], ulcerative colitis [HR=1.47,95%CI(1.29-1.68), *I²* =75.40%, *P<*0.001], idiopathic thrombocytopenia purpura [HR=1.45, 95% CI (1.15-1.83), *I²* = 51.40%, *P* = 0.002], thyroid dysfunction [HR=1.44, 95% CI (1.24-1.66), *I²* = 77.60%, *P<*0.001]. AD was hardly associated with an increased risk of multiple sclerosis [HR = 1.08, 95% CI (0.95-1.23, *I²* = 0.00%, *P* = 0.247 >0.05] (**Table 3**). A detailed forest map are shown in **Supplementary Figures 1**-**14**.

**Table 3:** Atopic dermatitis and risk of specific autoimmune disease.

Disease	NO	HR	95%CI	*I²*	*P*
Autoimmune disease	9	1.67	1.43-1.95	99.00%	<0.001
Specific autoimmune disease					
Celiac disease	9	2.12	1.89-2.38	80.90%	<0.001
Systemic lupus erythematosus	6	1.87	1.48-2.35	80.20%	<0.001
Psoriasis	6	3.39	2.17-5.31	97.20%	<0.001
Vitiligo	10	2.34	1.72-3.19	95.70%	<0.001
Thyroid dysfunction	8	1.44	1.24-1.66	77.60%	<0.001
Sjögren’s syndrome	4	2.08	1.45-3.00	77.50%	<0.001
Ankylosing sponylitis	4	1.65	1.30-2.09	48.60%	<0.001
Alopecia areata	8	7.80	4.39-13.86	97.80%	<0.001
Rheumatoid arthritis	7	1.47	1.39-1.56	0.00%	<0.001
Crohn disease	9	1.50	1.23-1.83	85.70%	<0.001
Multiple sclerosis	4	1.08	0.95-1.23	0.00%	0.247
Ulcerative colitis	9	1.47	1.29-1.68	75.40%	<0.001
Idiopathic thrombocytopenia purpura	5	1.45	1.15-1.83	51.40%	0.002

CI, confidence interval; HR, Hazard Ratio; NO, study number.

### Subgroup analysis

It is noteworthy that among the 26 selected pieces of literature, only nine explicitly proposed the HR value between AD and autoimmune diseases. Of these nine, three explicitly discussed the risk of children and autoimmune diseases, two discussed the risk of adults and autoimmune diseases, and the remaining four covered all age groups it was not possible to discern whether the age group was related.

There is a statistically significant correlation between individuals with AD aged ≤18 years and autoimmune disease[HR=1.49, 95% CI 1.31-1.70; *I²*=96.5%, *P<*0.001]. In the context of AD and specific autoimmune diseases, adolescents with AD exhibit an extreme susceptibility to the complication of psoriasis [HR=4.12, 95% CI (2.38-7.13); *I²*=98.3%, *P<*0.001], followed by coeliac disease [HR=2.08, 95% CI(1.91-2.26); *I²*=0.00%, *P*<0.001]. Furthermore, there is an increased susceptibility to juvenile arthritis [HR=1.36, 95% CI (1.20-1.54); *I²*=58.60%, *P<*0.001], vitiligo[HR=1.97,95% CI (1.47-2.64); *I²*=93.30%, *P<*0.001], idiopathic thrombocytopenia purpura [HR=1.39, 95% CI (1.10-1.76);*I²*=53.90%, *P*=0.006], and thyroid dysfunction [HR=1.60, 95% CI (1.08-2.36); *I²*=74.50%, *P*=0.018]. A detailed forest map are shown in **Supplementary Figures 14**, **15**.

In contrast, only two studies demonstrated a statistically significant correlation between atopic dermatitis and autoimmune disease in the AD population aged over 18 years [HR=1.75, 95% CI (1.12-2.72); *I²*=0.00%, *P*=0.014]. In the case of adult AD patients, there is an increased susceptibility to celiac disease [HR=2.60, 95% CI (1.61-4.20); *I²*=94.80%, *P<*0.001], Crohn’s disease [HR=1.88, 95% CI (1.52-2.31); *I²*=0.00%, *P<*0.001], IBD [HR=1.72, 95% CI (1.43-2.06); *I²*=26.30%, *P<*0.001], ulcerative colitis [HR=1.70, 95% CI (1.48-1.96); *I²*=0.00%, *P<*0.001], thyroid dysfunction [HR=1.43, 95% CI (1.28-1.70); *I²*=77.80%, *P<*0.001]. A detailed forest map are shown in **Supplementary Figures 14**, **16**.

Of the nine articles of atopic dermatitis and the risk of autoimmune disease, four addressed the risk of autoimmune disease with AD in women, and two addressed the risk of autoimmune disease in men with AD. Female patients with AD were more likely to have autoimmune diseases [HR=1.49, 95%CI(1.15-1.91); *I²*=98.8%, *P*=0.002]. A detailed forest map are shown in **Supplementary Figure 17**.

In subgroup analyses by study design, meta-analyses of Cross-sectional studies demonstrated a significant association between AD and an increased risk of autoimmune disease [HR=1.96, 95% CI (1.39-2.77); *I²*=90.8%, *P*<0.001], whereas meta-analyses of cohort studies showed a similar association between AD and the risk of autoimmune disease [HR=1.34, 95% (CI 1.22-1.48); *I²*=95.0%, *P*<0.001] (**Table 4**). A detailed forest maps are shown in **Supplementary Figure 18**.

**Table 4:** Subgroup analysis for the risk of autoimmune disease associated with atopic dermatitis.

Subgroups	NO	HR	95%CI	*I²*	*P*
Age					
≤18years	3	1.49	1.31-1.70	96.50%	<0.001
Type 1 diabetes	3	0.97	0.80-1.17	50.00%	0.740
Juvenile arthritis	6	1.36	1.20-1.54	58.60%	<0.001
Vitiligo	4	1.97	1.47-2.64	93.30%	<0.001
Thyroid dysfunction	4	1.60	1.08-2.36	74.50%	0.018
Idiopathic thrombocytopenia purpura	4	1.39	1.10-1.76	53.90%	0.006
Psoriasis	3	4.12	2.38-7.13	98.30%	<0.001
Celiac disease	3	2.08	1.91-2.26	0.00%	<0.001
>18years	2	1.75	1.12-2.72	0.00%	0.014
Thyroid dysfunction	3	1.43	1.28-1.70	77.80%	<0.001
Crohn disease	3	1.88	1.52-2.31	0.00%	<0.001
Celiac disease	3	2.60	1.61-4.20	94.80%	<0.001
Ulcerative colitis	3	1.70	1.48-1.96	0.00%	<0.001
IBD	3	1.72	1.43-2.06	26.30%	<0.001
Gender					
Women	4	1.49	1.15-1.91	98.80%	0.002
Men	2	1.31	1.25-1.38	0.00%	<0.001
Study type					
Cohort study	4	1.34	1.22-1.48	95.00%	<0.001
Cross-sectional study	4	1.96	1.39-2.77	90.80%	<0.001

CI, confidence interval; HR, Hazard Ratio; NO, study number.

### Publication bias

**Figure 3:**
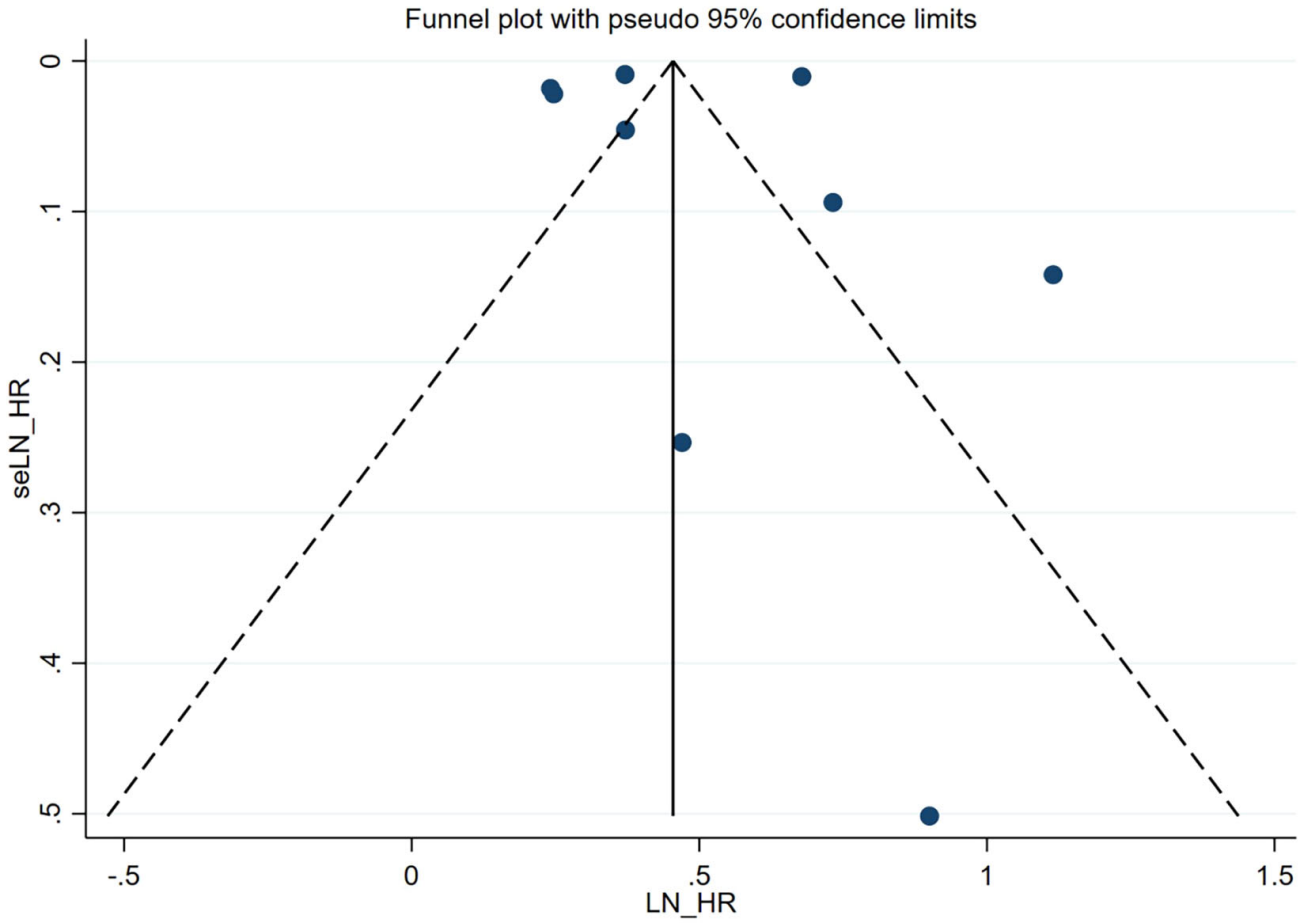
Funnel plot. Publication bias of the risk of atopic dermatitis associated with autoimmune diseases.

A visual inspection of the funnel plot did not reveal any evidence of significant publication bias in the assessment of the risk of comorbid autoimmune diseases in patients with atopic dermatitis. To formally evaluate publication bias, the Egger test was conducted. The results of this test confirmed the absence of statistically significant publication bias (*P* = 0.928). The funnel plot is presented in **Figure 3**.

## Discussion

### Main findings

The meta-analysis included a total of 26 studies providing a comprehensive assessment of the association between AD and the risk of developing autoimmune diseases. The results demonstrated a significant association between AD and an increased risk of autoimmune conditions across all age groups, including conditions such as coeliac disease, systemic lupus erythematosus, alopecia areata, rheumatoid arthritis, Crohn’s disease, ulcerative colitis and idiopathic thrombocytopenia purpura. Further subgroup analyses, including age, gender, and studies design (cohort vs case-control), revealed that the significant correlation between AD and elevated autoimmune disease risk was consistent across these different factors.

This large-scale, comprehensive meta-analysis comprehensively characterizes the heightened risk of a broad spectrum of autoimmune conditions in patients with atopic dermatitis, underscoring the importance of this relationship for clinical practice and patient management.

### Interpretation of findings

A previous review encompassed 14 studies that investigated the markedly elevated risk of selected autoimmune diseases in patients with AD (9). The findings indicated that AD can markedly increase the risk of autoimmune diseases, including systemic lupus erythematosus, vitiligo, coeliac disease, Crohn’s disease, ulcerative colitis, rheumatoid arthritis, and others (9). We conducted separate analyses of autoimmune disease incidence in both pediatric (≤18 years) and adult AD patient populations. The results demonstrated significant associations between AD and autoimmune disease incidence in both age groups. Notably, substantial heterogeneity (*I²*=96.5%) was observed in the pediatric AD analysis. Given the limited number of included studies (n=3, comprising two South Korean studies and one US study), we performed additional country-stratified analyses. These analyses revealed a marked reduction in heterogeneity following country stratification (**Supplementary Figure 19**), strongly suggesting that geographic variations (potentially including differences in diagnostic criteria, environmental factors, or genetic backgrounds) represent a key contributor to the initial high heterogeneity. These findings provide novel insights into the AD-autoimmune disease relationship while highlighting the importance of considering geographic factors in future multicenter studies.

While the previous review incorporated a study on both adults and children (30), it did not elaborate on the potential differences in the autoimmune disease risk between these two populations.

This is an important consideration, as a substantial body of literature has documented the high prevalence of AD in pediatric patients, with rates reaching up to 20% in children in high-income countries (2, 38, 39). Children with AD often experience disrupted sleep, daytime fatigue, and academic underperformance (40, 41), which can result in depressive and psychological symptoms (42). Furthermore, psychological stress can influence the progression and outcome of the autoimmune disease (43).

Furthermore, the relative immaturity of the adolescent immune system (44, 45) — characterized by incomplete development of secondary lymphoid organs and immature immunoregulatory networks—must be carefully considered. Notably, given that 70-80% of immune cells reside in the gut, intricate interactions exist among the gut microbiota, intestinal epithelium, and local mucosal immune system (44). In pediatric AD patients (particularly preschoolers), the developmental stage of the immune system(e.g., gut) differs substantially from adolescents. This immunological dynamism may profoundly influence the patterns of autoimmune comorbidity, necessitating age-stratified investigations (45).

Of particular significance, genomic studies have identified numerous shared genetic susceptibility factors between AD and autoimmune diseases. These include: (1) Specific HLA haplotypes (e.g., HLA-DR4, HLA-DQ8) that demonstrate established associations with both AD and multiple autoimmune disorders (46, 47); and (2) Variations in key epithelial barrier gene networks (e.g., FLG, SPINK5) that not only compromise skin barrier integrity but may also participate in systemic autoimmune responses through epigenetic regulation (48). Importantly, the expression patterns of these genetic factors in children may exhibit unique characteristics due to immune system immaturity, offering novel insights into the pathogenesis of autoimmune comorbidities in pediatric AD patients.

Additionally, given that the immune system of adolescents is not yet fully developed (49, 50), the potential for autoimmune complications in children with AD requires careful consideration and further investigation.

The findings of our study indicate that both adults and children with AD are susceptible to complications associated with autoimmune diseases. Interestingly, the results suggest that adolescents may experience a greater range of autoimmune disease-related complications compared to other age groups.

There is a possibility that children may be more susceptible to atopic dermatitis as a result of a number of factors. As individuals age, a strong immune-activating effect has been observed in the skin lesions of pediatric AD patients in response to Th2, Th9 and Th17, with elevated levels of Th2 and Th17 markers in the blood (51, 52). The prevailing view of pediatric AD is that it is driven primarily by Th2 signaling.Th2 cells secrete cytokines such as IL-4 and IL-13, which not only mediate IgE class switching in B cells but also upregulate FcϵRI receptor expression on mast cells/basophils, thereby enhancing IgE-mediated immune responses (53, 54). Elevated IgE levels have been identified as a potential risk factor for the development of allergic diseases (55, 56). Following birth and throughout childhood, when the gut microbial system is not yet fully developed, the natural maturation of the immune system is inhibited, and Tregs fail to mature sufficiently to regulate certain balances, such as those between Th1 and Th2 (57). Furthermore, the human skin barrier is structurally and functionally immature at birth. This is evidenced by elevated skin surface pH, lower lipid levels, and lower resistance to chemicals and pathogens (58). It is evident that these factors have the potential to increase the incidence of AD in children. Furthermore, children are more susceptible to the influence of family members. For example, there is a clear association between the mother and the child’s AD disease. It has been documented that Maternal diet during pregnancy、timing of complementary food introduction、prenatal/early-life probiotic/prebiotic supplementation (59, 60),the status of maternal intestinal flora (61), maternal antibiotic use during pregnancy (62) and even maternal constipation (63) seems to be related to the pathophysiological development of atopic dermatitis in children. As will be discussed subsequently, it seems reasonable to posit that the fact that children are undergoing growth and that their immune systems are inherently unstable, coupled with the fact that they are also susceptible to genetic influences that are contributing to an increase in the prevalence of childhood conditions, is the reason behind this phenomenon.

In comparison to the immune system of a child, the adult immune system has matured and is capable of effectively regulating the body’s immune response. Similarly, adults are susceptible to increased morbidity as a result of prolonged stress, encompassing familial, occupational, and social stressors (64).The prevalence of AD in adults is associated with a significant financial burden on healthcare systems, including increased direct and indirect costs of care and lost productivity (65). It is evident that greater attention should be paid to the impact of AD on our lives, and to the disease itself.

Lastly, the study reveals that women with AD are at a higher risk of developing autoimmune diseases compared to men. This phenomenon may be related to the differential effects of sex hormones on Th2/Treg and Th1/Th17 cell activities (66–69). These comprehensive findings underscore the complex interplay between AD, the immune system, the gut microbiome, and various genetic, hormonal, and environmental factors in modulating the risk of autoimmune diseases, particularly in the adolescent population.

### Implications and limitations

The principal strength of our meta-analysis was the inclusion of 26 pertinent observational studies, thereby ensuring a robust assessment of the association between atopic dermatitis and the risk of autoimmune disease. The large combined sample size enabled an effective assessment of this association. Our findings indicate that atopic dermatitis is a significant risk factor for autoimmune and other diseases. It is noteworthy that the analysis included both gender and article type, effectively managing confounding factors and improving the reliability of the conclusions.

It is important to consider the limitations of the meta-analysis. Firstly, the present study only considered articles published during the ten years 2014-2024. However, it would be possible to extend the period under review in future studies, thereby increasing the sample size for analysis. Secondly, although the fully adjusted estimated effect with 95% CI was extracted, adjusted confounders (e.g. medication use and socioeconomic status, etc.) were not consistent across the included studies. Thirdly, when discussing subgroup analyses of age, we undertook a strict differentiation between the discussion of samples of children and adults. As the inclusion of the majority of the literature was straightforward in discussing each age group together, the number of studies examining the onset of autoimmune disease in children versus adults with AD alone was limited. Further studies are required to analyze and discuss this issue in greater depth.

Fourthly, our analysis demonstrates a significant association between AD and autoimmune diseases (rather than specific autoimmune diseases), despite considerable heterogeneity (*I²*=96.5%). The limited number of eligible studies meeting our inclusion criteria (n=3) precluded further subgroup analyses to investigate potential sources of this heterogeneity. To address this limitation, we conducted supplementary analyses in **Table 4** examining associations between ≤18-years AD patients and specific autoimmune diseases, including type 1 diabetes, juvenile idiopathic arthritis, and autoimmune thyroiditis. These analyses revealed statistically significant associations for all evaluated conditions (all *P*<0.05), except for type 1 diabetes which showed no significant correlation. While these findings hold potential clinical relevance, the interpretation requires caution due to the limited number of primary studies and substantial heterogeneity, underscoring the need for validation through larger-scale, well-designed prospective studies.

Finally, approximately 75% of studies included in our analysis employed retrospective designs, which indeed introduces limitations, particularly impacting the reliability of causal inferences for children and adolescents (≤18 years). While our meta-analysis demonstrated significant associations between AD (especially childhood-onset) and various autoimmune diseases, the predominantly retrospective nature of included studies suggests these findings should be interpreted as suggestive rather than conclusive evidence. The conclusion explicitly emphasizes the need for cautious interpretation of results for the ≤18 years population and strongly advocates for validation through future prospective cohort studies.

### Future prospects

Based on current Mendelian randomization evidence and the meta-analysis findings from our study, there exists a clear epidemiological association and potential causal relationship between AD and autoimmune diseases. Building upon these discoveries, future research should focus on elucidating the precise pathogenic mechanisms linking these conditions and exploring clinical translation. Regarding mechanistic investigations, the ‘epithelial-immune axis’ hypothesis provides an important framework for understanding this association: persistent skin barrier dysfunction in AD patients may trigger systemic immune dysregulation through the release of epithelial-derived alarmins such IL-33, ultimately leading to aberrant activation of autoimmune responses. This hypothesis can be tested including but not limited to: (1) establishing an MC903-induced AD animal model followed by long-term (≥6 months) follow-up observations to determine whether spontaneous autoimmune manifestations develop; (2) employing organoid co-culture systems to simulate epithelial-immune cell interactions; and (3) utilizing humanized mouse models to investigate the role of alarmins such as IL-33 (54, 70–72).

At the clinical practice level, we recommend implementing risk-stratified management for AD patients across both adult and pediatric populations, with particular surveillance recommended for comorbid autoimmune conditions including alopecia areata, psoriasis, celiac disease, rheumatoid arthritis, and ulcerative colitis (see **Table 5** for detailed stratification criteria). Particular attention should be given to patients with either a family history of autoimmune diseases or refractory dermatitis. Furthermore, conducting randomized controlled studies to compare the therapeutic efficacy between conventional immunosuppressants (e.g., methotrexate 10–15 mg/week) and novel biologics (e.g., anti-IL-4Rα monoclonal antibodies) in AD patients with comorbid autoimmune diseases will provide crucial evidence-based guidance for clinical decision-making.

**Table 5:** Evidence-Based Screening Recommendations for Autoimmune Diseases in Atopic Dermatitis (AD) Patients.

Autoimmune Disease	Target AD Population	Gender	Age	Screening Tests	Note	References
Alopecia Areata	•AD patients develop circular/round patches of hair loss.	No difference.	No difference.	Trichoscopy + anti-hair follicle antibodies	•Clinical symptoms of different diseases need to be differentiated (e.g. Alopecia Areata, discoid lupus erythematosus, etc.).	(73–75)
Psoriasis	•Treatment-resistant flexural AD • Nail pitting/onycholysis	No difference.	A higher incidence of psoriasis is observed in pediatric populations (≤18 years).	Dermatologic examination, HLA-Cw6 testing	•Recording the response of skin lesions to climate (psoriasis worsens in winter, AD worsens in summer) •Distinct treatment regimens are required for pregnancy and lactation periods	(76–79)
Celiac Disease	• AD with chronic diarrhea/weight loss.	Women are significantly higher than men.	About 60-70% of cases are diagnosed in childhood, peaking at ages 1-3 (after gluten introduction) and adolescence.	Anti-tTG-IgA + anti-EMA antibodies	•Gluten-free diet improves some AD symptoms.	(80–82)
Rheumatoid Arthritis	•With arthralgia.	No difference.	Adults ≥30years	Anti-CCP + RF factor	•Patients with arthralgia need to be referred to rheumatology.	(83–85)
Systemic Lupus Erythematosus	• Photosensitivity exacerbating AD • Malar rash	Women with AD are significantly higher than men.	No difference.	ANA, anti-dsDNA antibodies, complement C3/C4	•The difference in rash (SLE butterfly erythema Vs AD eczema) also requires biopsy to identify	(86–89)
Ulcerative Colitis	•AD with recurrent abdominal pain/hematochezia.	No difference.	The incidence rate is higher in adults.	Fecal calprotectin + colonoscopy	•There was a 50% increased risk of bowel cancer in people with specific dermatitis for up to 10 years and UC.	(90–92)
Thyroid Dysfunction	• Postpartum (within 1 year)	Females with AD are significantly higher than men.	No difference.	TSH + FT4 + anti-TPO antibodies	•Test TSH+ TPO-AB before 8 weeks’ gestation (even if normal), TPO antibody positive: 16–20 weeks’ gestation TSH review. •In children, growth monitoring is essential, with distinct TSH cutoffs from adults.	(93–96)

## Conclusion

This comprehensive meta-analysis assessed the risk of autoimmune diseases in patients with atopic dermatitis. The results demonstrated a significant association between AD and an increased risk of developing a broad range of autoimmune conditions, including alopecia areata, coeliac disease, Sjögren’s syndrome, ulcerative colitis, and others.

These findings underscore the importance for clinicians to have a high index of suspicion for potential autoimmune diseases when evaluating and managing patients diagnosed with atopic dermatitis.

The strong link between AD and elevated autoimmune disease risk highlighted by this meta-analysis has critical implications for clinical practice. Clinicians should consider screening for and closely monitoring AD patients for the development of associated autoimmune conditions to enable early intervention and improved patient outcomes.

These comprehensive meta-analysis results emphasize the need for a multidisciplinary, integrated approach to the management of atopic dermatitis, incorporating vigilance for comorbid autoimmune diseases. This will be essential for providing optimal, holistic care for individuals suffering from this complex, multisystem condition.

## Data Availability

The original contributions presented in the study are included in the article/**Supplementary Material**. Further inquiries can be directed to the corresponding authors.
