# Supplementary material for: Atopic dermatitis and risk of autoimmune diseases: a systematic review and meta-analysis
Source: Front Immunol. 2025 Jun 12;16:1539997. doi: 10.3389/fimmu.2025.1539997 (PMC12198157; doi:10.3389/fimmu.2025.1539997)
Supplement: Supplementary file 2 [file SupplementaryFile2.docx]

**[Supplementary](https://mc.manuscriptcentral.com/bjd?DOWNLOAD=TRUE&PARAMS=xik_6h7Yg4S3pRuK9mArvfiXi7JeDZrkUe3SNCZZueAgbTXqNXs9aeAmVMKHzL1gE2HZnCbW2ZgGRvwxA8eANqS6kzxJ6dWKDw3VXuwfZysTq2LB6gGmNymh2V1NMr41j82QbpmnRpu9K7VsFQ5u2m4r4DkhkWoJAmq5pEmTfWMvHoNqNRzwc9pDgDURbfvjc7jAX5TD7) Table 1-3: Details of the Literature Search Strategy**

**Table 1** PubMed

| Search | Query | Results |
| --- | --- | --- |
| #1 | (((((((((((((((((((((((((((((((((((((((((((((((((Addison Disease[Title/Abstract]) OR (Anemia, Hemolytic, Autoimmune[Title/Abstract])) OR (Anti-Glomerular Basement Membrane Disease[Title/Abstract])) OR (Anti-Neutrophil Cytoplasmic Antibody-Associated Vasculitis[Title/Abstract])) OR (Churg-Strauss Syndrome[Title/Abstract])) OR (Granulomatosis with Polyangiitis[Title/Abstract])) OR (Microscopic Polyangiitis[Title/Abstract])) OR (Antiphospholipid Syndrome[Title/Abstract])) OR (Arthritis, Juvenile[Title/Abstract])) OR (Arthritis, Rheumatoid[Title/Abstract])) OR (Felty Syndrome[Title/Abstract])) OR (Rheumatoid Vasculitis[Title/Abstract])) OR (Sjogren's Syndrome[Title/Abstract])) OR (Still's Disease, Adult-Onset[Title/Abstract])) OR (Autoimmune Diseases of the Nervous System[Title/Abstract])) OR (Anti-N-Methyl-D-Aspartate Receptor Encephalitis[Title/Abstract])) OR (Demyelinating Autoimmune Diseases, CNS[Title/Abstract])) OR (Myasthenia Gravis[Title/Abstract])) OR (Nervous System Autoimmune Disease, Experimental[Title/Abstract])) OR (Polyradiculoneuropathy[Title/Abstract])) OR (Stiff-Person Syndrome[Title/Abstract])) OR (Uveomeningoencephalitic Syndrome[Title/Abstract])) OR (Vasculitis, Central Nervous System[Title/Abstract])) OR (Autoimmune Hypophysitis[Title/Abstract])) OR (Autoimmune Lymphoproliferative Syndrome[Title/Abstract])) OR (Autoimmune Pancreatitis[Title/Abstract])) OR (Birdshot Chorioretinopathy[Title/Abstract])) OR (Dermatitis Herpetiformis[Title/Abstract])) OR (Diabetes Mellitus, Type 1[Title/Abstract])) OR (Glomerulonephritis, IGA[Title/Abstract])) OR (Glomerulonephritis, Membranous[Title/Abstract])) OR (Graves Disease[Title/Abstract])) OR (Graves Ophthalmopathy[Title/Abstract])) OR (Hepatitis, Autoimmune[Title/Abstract])) OR (Immunoglobulin G4-Related Disease[Title/Abstract])) OR (Latent Autoimmune Diabetes in Adults[Title/Abstract])) OR (Linear IgA Bullous Dermatosis[Title/Abstract])) OR (Lupus Erythematosus, Systemic[Title/Abstract])) OR (Lupus Nephritis[Title/Abstract])) OR (Lupus Vasculitis, Central Nervous System[Title/Abstract])) OR (Ophthalmia, Sympathetic[Title/Abstract])) OR (Pemphigoid, Bullous[Title/Abstract])) OR (Pemphigus[Title/Abstract])) OR (Polyendocrinopathies, Autoimmune[Title/Abstract])) OR (Purpura, Thrombocytopenic, Idiopathic[Title/Abstract])) OR (Thyroiditis, Autoimmune[Title/Abstract])) OR (Hashimoto Disease[Title/Abstract])) OR (Postpartum Thyroiditis[Title/Abstract])) OR (Undifferentiated Connective Tissue Diseases[Title/Abstract])) OR ( Celiac Disease[Title/Abstract])) OR ((((Autoimmune Disease*[Title/Abstract]) OR (Immune System Disease*[Title/Abstract])) OR (Autoantibodies[Title/Abstract])) OR (Autoimmunity[Title/Abstract])) | 249625 |
| #2 | (atopic dermatitis[Title/Abstract])) OR (atopy[Title/Abstract]) | 42295 |
| #3 | (((((((((((((((((((((((((((((((((((((((((((((((((Addison Disease[Title/Abstract]) OR (Anemia, Hemolytic, Autoimmune[Title/Abstract])) OR (Anti-Glomerular Basement Membrane Disease[Title/Abstract])) OR (Anti-Neutrophil Cytoplasmic Antibody-Associated Vasculitis[Title/Abstract])) OR (Churg-Strauss Syndrome[Title/Abstract])) OR (Granulomatosis with Polyangiitis[Title/Abstract])) OR (Microscopic Polyangiitis[Title/Abstract])) OR (Antiphospholipid Syndrome[Title/Abstract])) OR (Arthritis, Juvenile[Title/Abstract])) OR (Arthritis, Rheumatoid[Title/Abstract])) OR (Felty Syndrome[Title/Abstract])) OR (Rheumatoid Vasculitis[Title/Abstract])) OR (Sjogren's Syndrome[Title/Abstract])) OR (Still's Disease, Adult-Onset[Title/Abstract])) OR (Autoimmune Diseases of the Nervous System[Title/Abstract])) OR (Anti-N-Methyl-D-Aspartate Receptor Encephalitis[Title/Abstract])) OR (Demyelinating Autoimmune Diseases, CNS[Title/Abstract])) OR (Myasthenia Gravis[Title/Abstract])) OR (Nervous System Autoimmune Disease, Experimental[Title/Abstract])) OR (Polyradiculoneuropathy[Title/Abstract])) OR (Stiff-Person Syndrome[Title/Abstract])) OR (Uveomeningoencephalitic Syndrome[Title/Abstract])) OR (Vasculitis, Central Nervous System[Title/Abstract])) OR (Autoimmune Hypophysitis[Title/Abstract])) OR (Autoimmune Lymphoproliferative Syndrome[Title/Abstract])) OR (Autoimmune Pancreatitis[Title/Abstract])) OR (Birdshot Chorioretinopathy[Title/Abstract])) OR (Dermatitis Herpetiformis[Title/Abstract])) OR (Diabetes Mellitus, Type 1[Title/Abstract])) OR (Glomerulonephritis, IGA[Title/Abstract])) OR (Glomerulonephritis, Membranous[Title/Abstract])) OR (Graves Disease[Title/Abstract])) OR (Graves Ophthalmopathy[Title/Abstract])) OR (Hepatitis, Autoimmune[Title/Abstract])) OR (Immunoglobulin G4-Related Disease[Title/Abstract])) OR (Latent Autoimmune Diabetes in Adults[Title/Abstract])) OR (Linear IgA Bullous Dermatosis[Title/Abstract])) OR (Lupus Erythematosus, Systemic[Title/Abstract])) OR (Lupus Nephritis[Title/Abstract])) OR (Lupus Vasculitis, Central Nervous System[Title/Abstract])) OR (Ophthalmia, Sympathetic[Title/Abstract])) OR (Pemphigoid, Bullous[Title/Abstract])) OR (Pemphigus[Title/Abstract])) OR (Polyendocrinopathies, Autoimmune[Title/Abstract])) OR (Purpura, Thrombocytopenic, Idiopathic[Title/Abstract])) OR (Thyroiditis, Autoimmune[Title/Abstract])) OR (Hashimoto Disease[Title/Abstract])) OR (Postpartum Thyroiditis[Title/Abstract])) OR (Undifferentiated Connective Tissue Diseases[Title/Abstract])) OR ( Celiac Disease[Title/Abstract])) OR ((((Autoimmune Disease*[Title/Abstract]) OR (Immune System Disease*[Title/Abstract])) OR (Autoantibodies[Title/Abstract])) OR (Autoimmunity[Title/Abstract])) AND (atopic dermatitis[Title/Abstract])) OR (atopy[Title/Abstract]) | 1069 |

**Table 2** Cochrane Library

| **Search** | **Query** | **Results** |
| --- | --- | --- |
| #1 | (atopic eczema):ti,ab,kw OR (atopic dermatitis):ti,ab,kw OR (atopy):ti,ab,kw | 7527 |
| #2 | (Autoimmune Disease*):ti,ab,kw OR (Immune System Disease*):ti,ab,kw OR (Autoantibodies):ti,ab,kw OR (Autoimmunity):ti,ab,kw | 16906 |
| #3 | (Addison Disease):ti,ab,kw OR (Anemia, Hemolytic, Autoimmune):ti,ab,kw OR (Anti-Glomerular Basement Membrane Disease):ti,ab,kw OR (Anti-Neutrophil Cytoplasmic Antibody-Associated Vasculitis):ti,ab,kw OR (Churg-Strauss Syndrome):ti,ab,kw | 653 |
| #4 | (Granulomatosis with Polyangiitis):ti,ab,kw OR (Microscopic Polyangiitis):ti,ab,kw OR (Antiphospholipid Syndrome):ti,ab,kw OR (Arthritis, Juvenile):ti,ab,kw OR (Arthritis, Rheumatoid):ti,ab,kw | 20235 |
| #5 | (Felty Syndrome):ti,ab,kw OR (Rheumatoid Vasculitis):ti,ab,kw OR (Sjogren's Syndrome):ti,ab,kw OR (Still's Disease, Adult-Onset):ti,ab,kw OR (Autoimmune Diseases of the Nervous System):ti,ab,kw | 1596 |
| #6 | (Anti-N-Methyl-D-Aspartate Receptor Encephalitis):ti,ab,kw OR (Demyelinating Autoimmune Diseases, CNS):ti,ab,kw OR (Myasthenia Gravis):ti,ab,kw OR (Nervous System Autoimmune Disease, Experimental):ti,ab,kw OR (Polyradiculoneuropathy):ti,ab,kw | 1319 |
| #7 | (Stiff-Person Syndrome):ti,ab,kw OR (Uveomeningoencephalitic Syndrome):ti,ab,kw OR (Vasculitis, Central Nervous System):ti,ab,kw OR (Autoimmune Hypophysitis):ti,ab,kw OR (Autoimmune Lymphoproliferative Syndrome):ti,ab,kw | 135 |
| #8 | (Autoimmune Pancreatitis):ti,ab,kw OR (Birdshot Chorioretinopathy):ti,ab,kw OR (Dermatitis Herpetiformis):ti,ab,kw OR (Diabetes Mellitus, Type 1):ti,ab,kw OR (Glomerulonephritis, IGA):ti,ab,kw | 43426 |
| #9 | (Glomerulonephritis, Membranous):ti,ab,kw OR (Graves Disease):ti,ab,kw OR (Graves Ophthalmopathy):ti,ab,kw OR (Hepatitis, Autoimmune):ti,ab,kw OR (Immunoglobulin G4 Related Disease):ti,ab,kw | 2293 |
| #10 | (Latent Autoimmune Diabetes in Adults):ti,ab,kw OR (Linear IgA Bullous Dermatosis):ti,ab,kw OR (Lupus Erythematosus, Systemic):ti,ab,kw OR (Lupus Nephritis):ti,ab,kw OR (Lupus Vasculitis, Central Nervous System):ti,ab,kw | 3877 |
| #11 | (Ophthalmia, Sympathetic):ti,ab,kw OR (Pemphigoid, Bullous):ti,ab,kw OR (Pemphigus):ti,ab,kw OR (Polyendocrinopathies, Autoimmune):ti,ab,kw OR (Purpura, Thrombocytopenic, Idiopathic):ti,ab,kw | 1282 |
| #12 | (Thyroiditis, Autoimmune):ti,ab,kw OR (Hashimoto Disease):ti,ab,kw OR (Postpartum Thyroiditis):ti,ab,kw OR (Undifferentiated Connective Tissue Diseases):ti,ab,kw OR (Celiac Disease):ti,ab,kw | 884 |
| #13 | #2 or #3 or #4 or #5 or #6 or #7 or #8 or #9 or #10 or #11 or #12 | 84686 |
| #14 | #13 and #1 | 286 |

**Table 3** Embase

| **Search** | **Query** | **Items found** |
| --- | --- | --- |
| #1 | 'autoimmune disease*' OR 'immune system disease*' OR 'autoantibodies'/exp OR 'autoantibodies' OR 'autoimmunity'/exp OR 'autoimmunity' OR 'addison disease':ab,ti OR 'anemia, hemolytic, autoimmune':ab,ti OR 'anti-glomerular basement membrane disease':ab,ti OR 'anti-neutrophil cytoplasmic antibody-associated vasculitis':ab,ti OR 'churg-strauss syndrome':ab,ti OR 'granulomatosis with polyangiitis':ab,ti OR 'microscopic polyangiitis':ab,ti OR 'antiphospholipid syndrome':ab,ti OR 'arthritis, juvenile':ab,ti OR 'arthritis, rheumatoid':ab,ti OR 'felty syndrome':ab,ti OR 'rheumatoid vasculitis':ab,ti | 552115 |
| #2 | 'sjogrens syndrome' OR 'stills disease, adult-onset' OR 'autoimmune diseases of the nervous system'/exp OR 'autoimmune diseases of the nervous system' OR 'anti-n-methyl-d-aspartate receptor encephalitis'/exp OR 'anti-n-methyl-d-aspartate receptor encephalitis' OR 'demyelinating autoimmune diseases, cns'/exp OR 'demyelinating autoimmune diseases, cns' OR 'myasthenia gravis'/exp OR 'myasthenia gravis' OR 'nervous system autoimmune disease, experimental'/exp OR 'nervous system autoimmune disease, experimental' OR 'polyradiculoneuropathy'/exp OR 'polyradiculoneuropathy' OR 'stiff-person syndrome'/exp OR 'stiff-person syndrome' OR 'uveomeningoencephalitic syndrome'/exp OR 'uveomeningoencephalitic syndrome' OR 'vasculitis, central nervous system'/exp OR 'vasculitis, central nervous system' OR 'autoimmune hypophysitis'/exp OR 'autoimmune hypophysitis' OR 'autoimmune lymphoproliferative syndrome'/exp OR 'autoimmune lymphoproliferative syndrome' OR 'autoimmune pancreatitis':ab,ti OR 'birdshot chorioretinopathy':ab,ti OR 'dermatitis herpetiformis':ab,ti OR 'diabetes mellitus, type 1':ab,ti OR 'glomerulonephritis, iga':ab,ti OR 'glomerulonephritis, membranous':ab,ti OR 'graves disease':ab,ti OR 'graves ophthalmopathy':ab,ti OR 'hepatitis, autoimmune':ab,ti OR 'immunoglobulin g4-related disease':ab,ti OR 'latent autoimmune diabetes in adults':ab,ti OR 'linear iga bullous dermatosis':ab,ti OR 'lupus erythematosus, systemic':ab,ti OR 'lupus nephritis':ab,ti OR 'lupus vasculitis, central nervous system':ab,ti OR 'ophthalmia, sympathetic':ab,ti OR 'pemphigoid, bullous':ab,ti OR 'pemphigus':ab,ti OR 'polyendocrinopathies, autoimmune':ab,ti OR 'purpura, thrombocytopenic, idiopathic':ab,ti OR 'thyroiditis, autoimmune':ab,ti OR 'hashimoto disease':ab,ti OR 'postpartum thyroiditis':ab,ti OR 'undifferentiated connective tissue diseases':ab,ti OR ' Celiac Disease':ab,ti | 161286 |
| #3 | #1 or #2 | 659770 |
| #4 | 'atopic dermatitis'/exp OR 'atopic dermatitis' OR 'atopy'/exp OR 'atopy' | 167425 |
| #5 | #3 or #4 | 6477 |
