# Supplementary material for: Atopic dermatitis and risk of autoimmune diseases: a systematic review and meta-analysis
Source: Front Immunol. 2025 Jun 12;16:1539997. doi: 10.3389/fimmu.2025.1539997 (PMC12198157; doi:10.3389/fimmu.2025.1539997)

WeChat conversation between Hongli Wang and Min Chen about the research

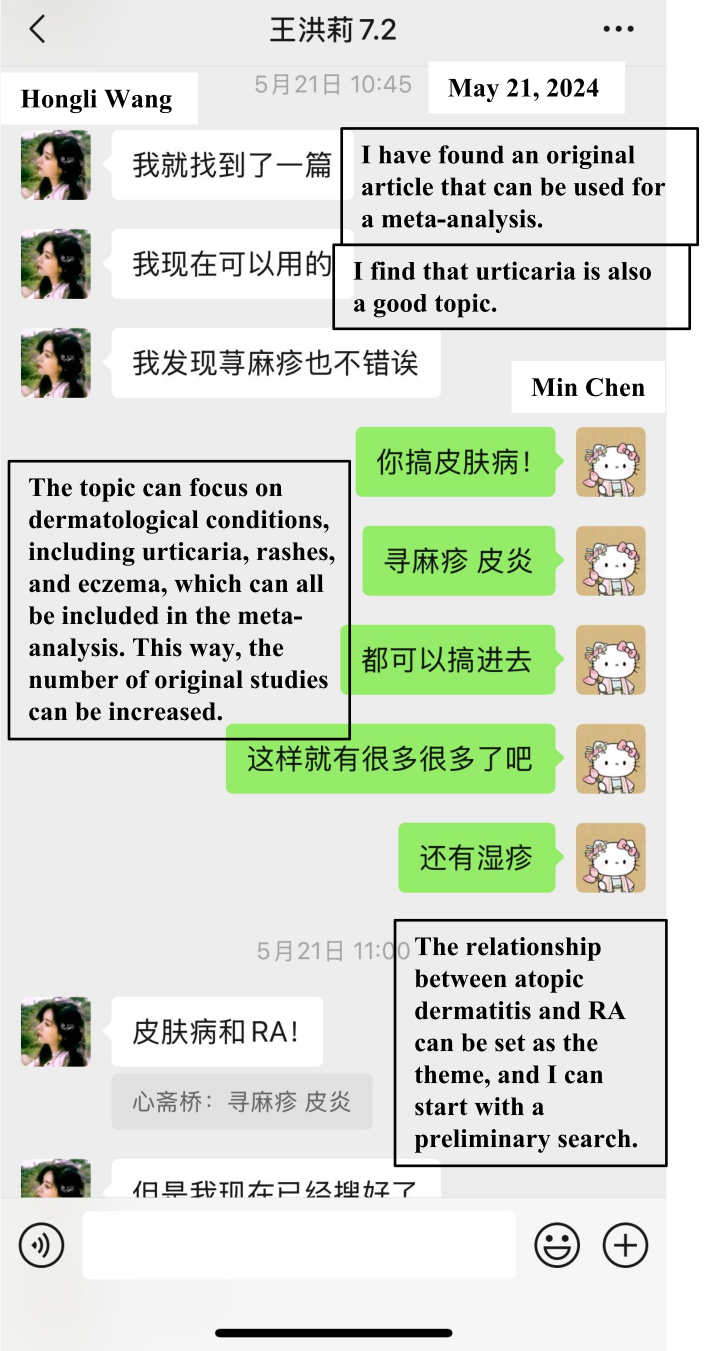

WeChat conversation between Hongli Wang and Min Chen about the research

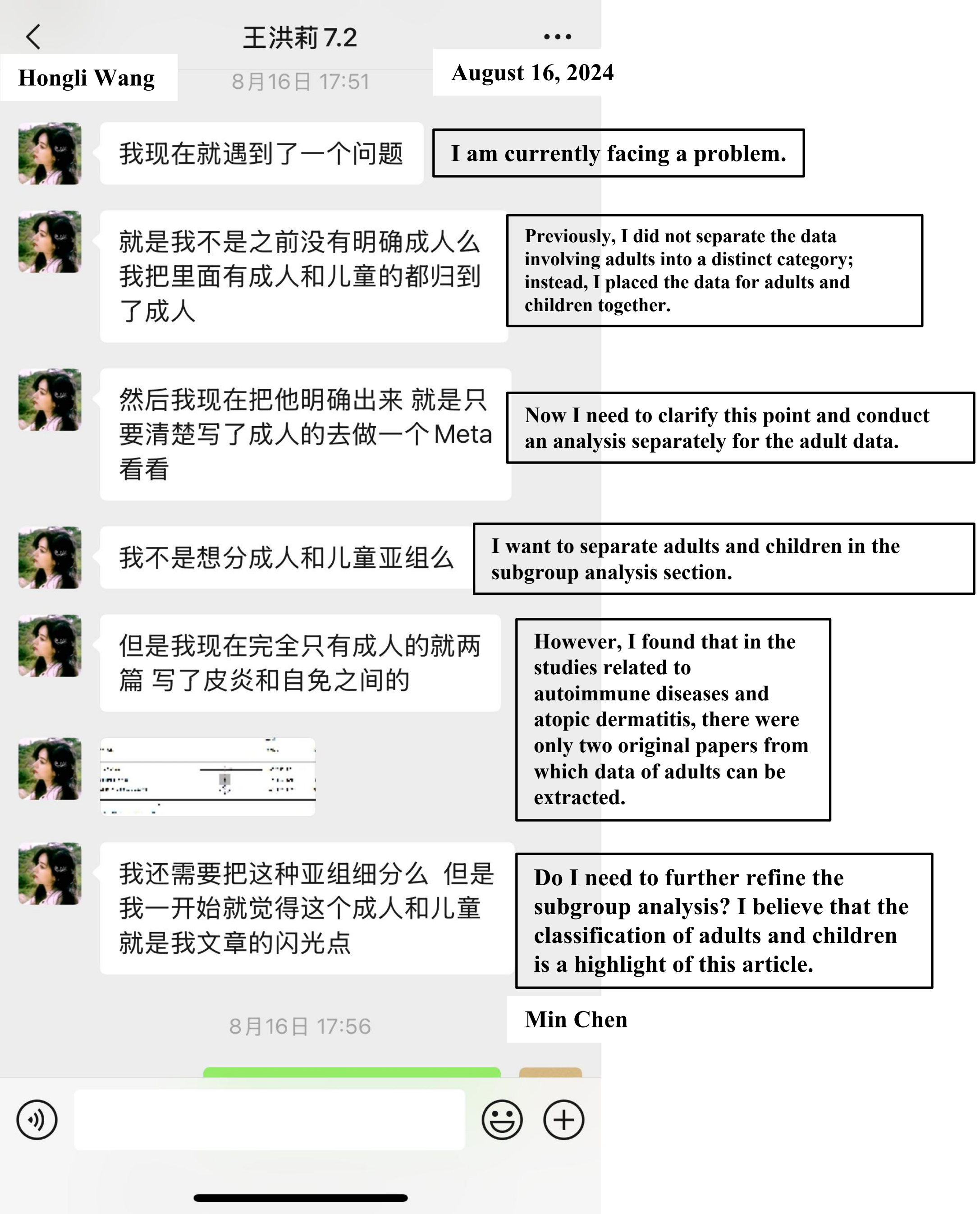

WeChat conversation between Hongli Wang and Min Chen about the research

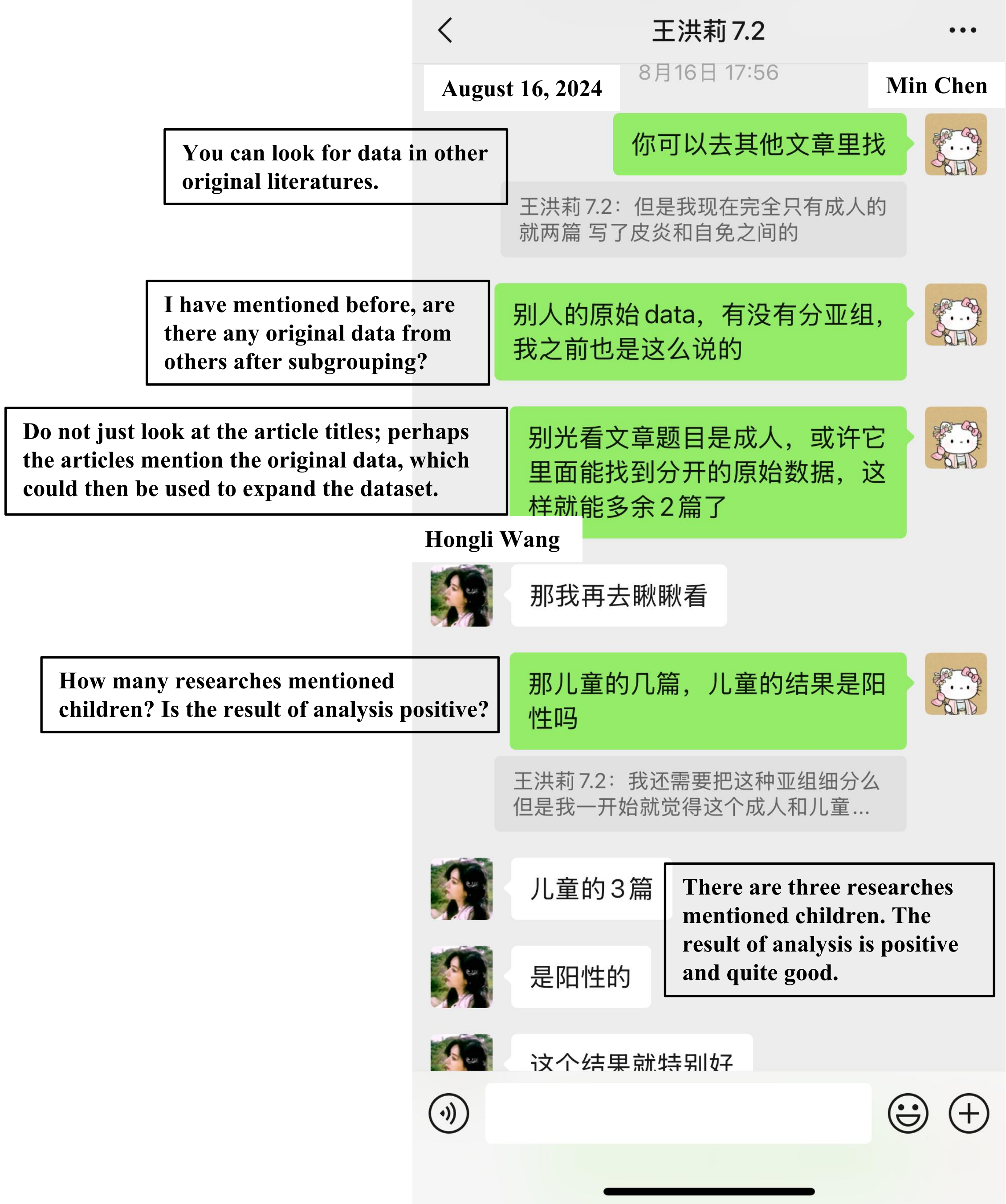

WeChat conversation between Hongli Wang and Min Chen about the research

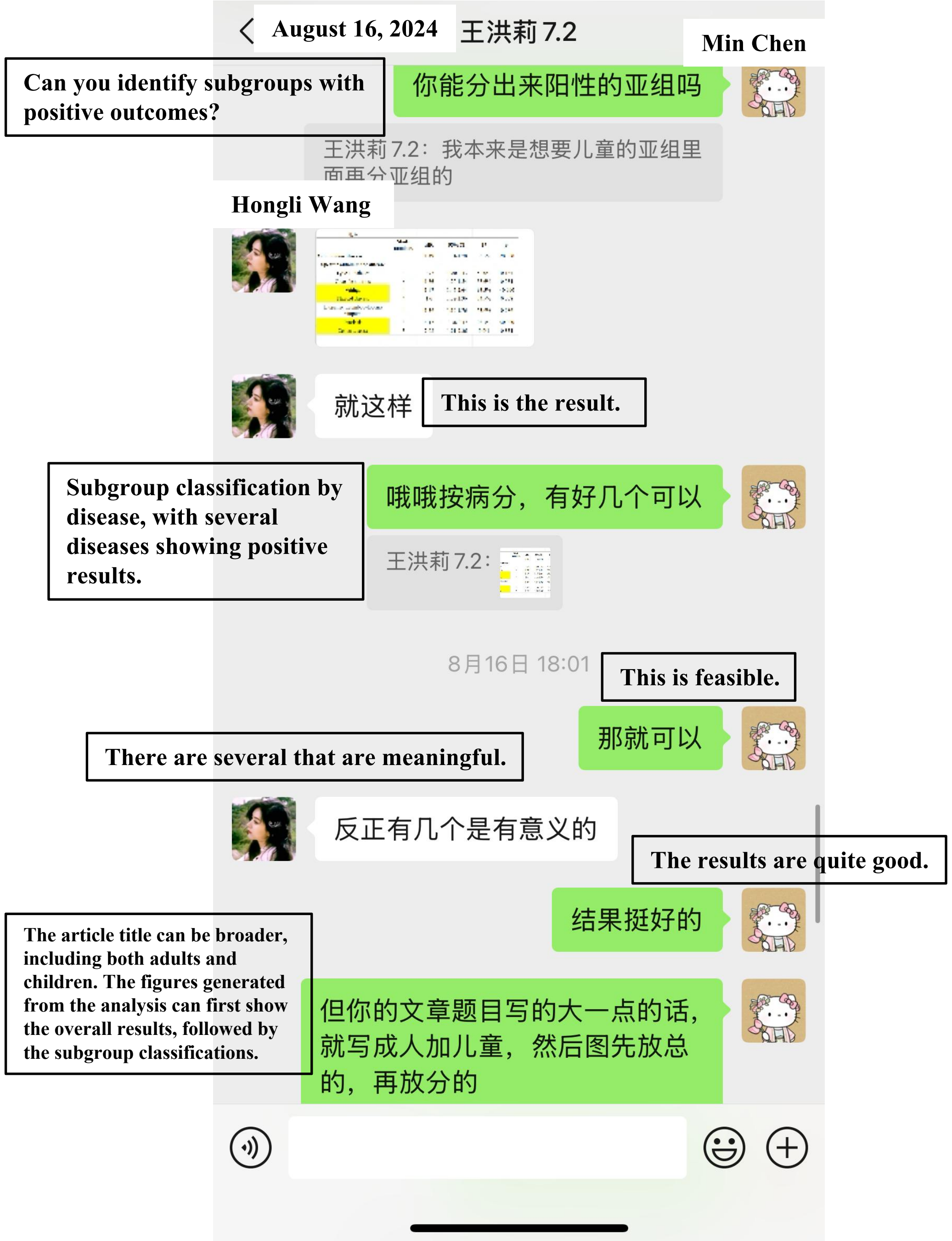

WeChat conversation between Hongli Wang and Min Chen about the research

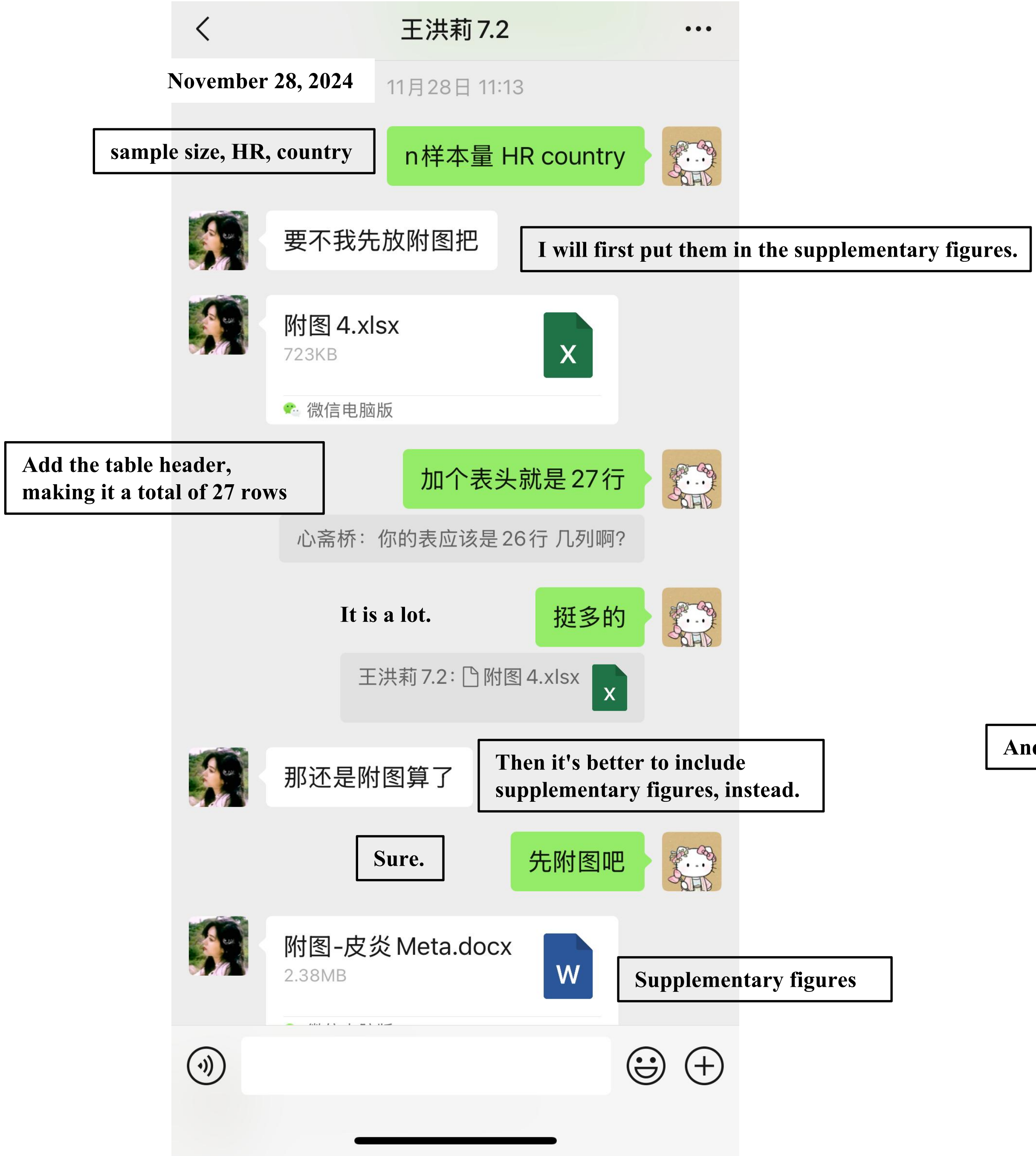

WeChat conversation between Hongli Wang and Min Chen about the research

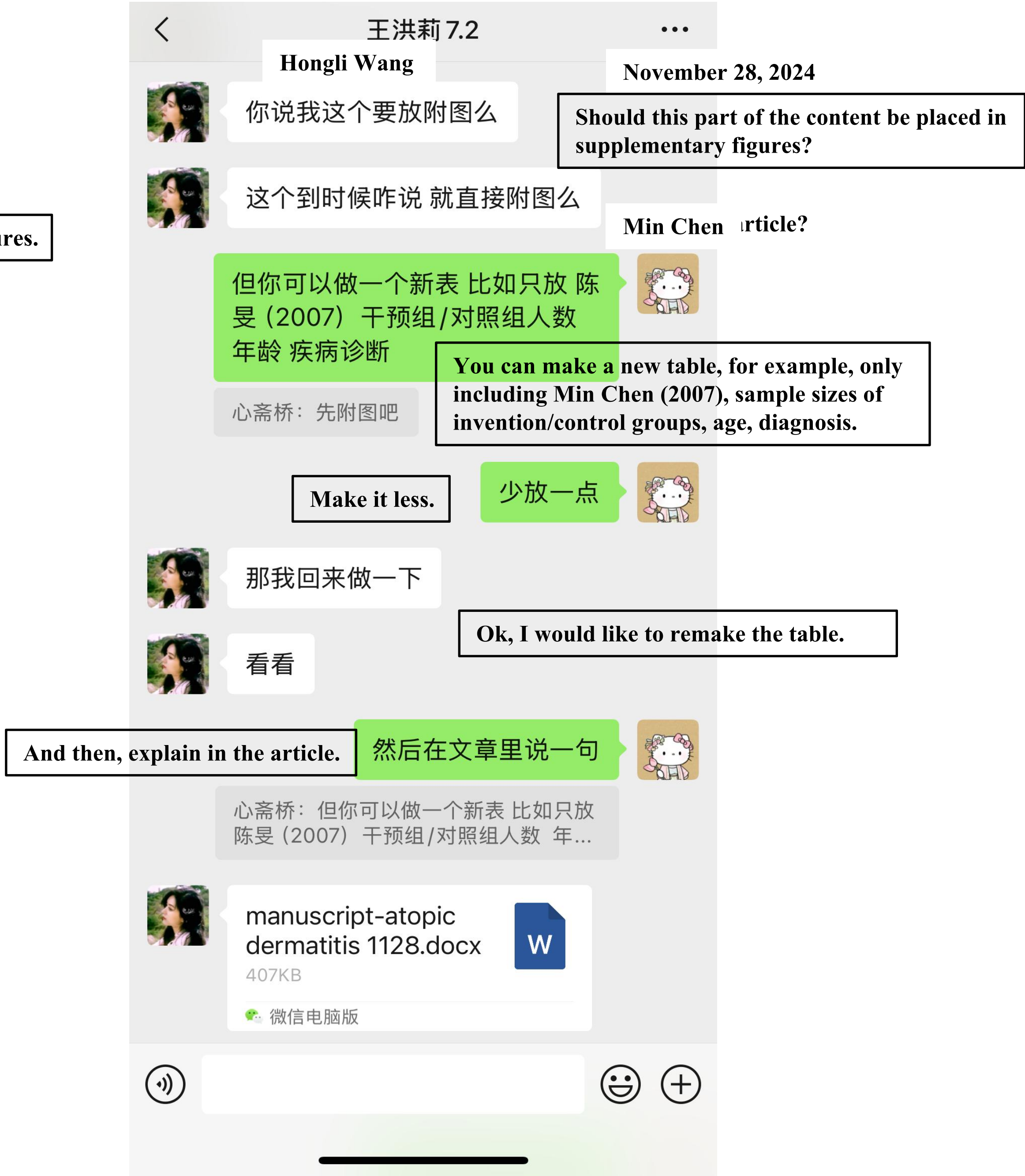

WeChat conversation between Hongli Wang and Tengyue Wang about the research

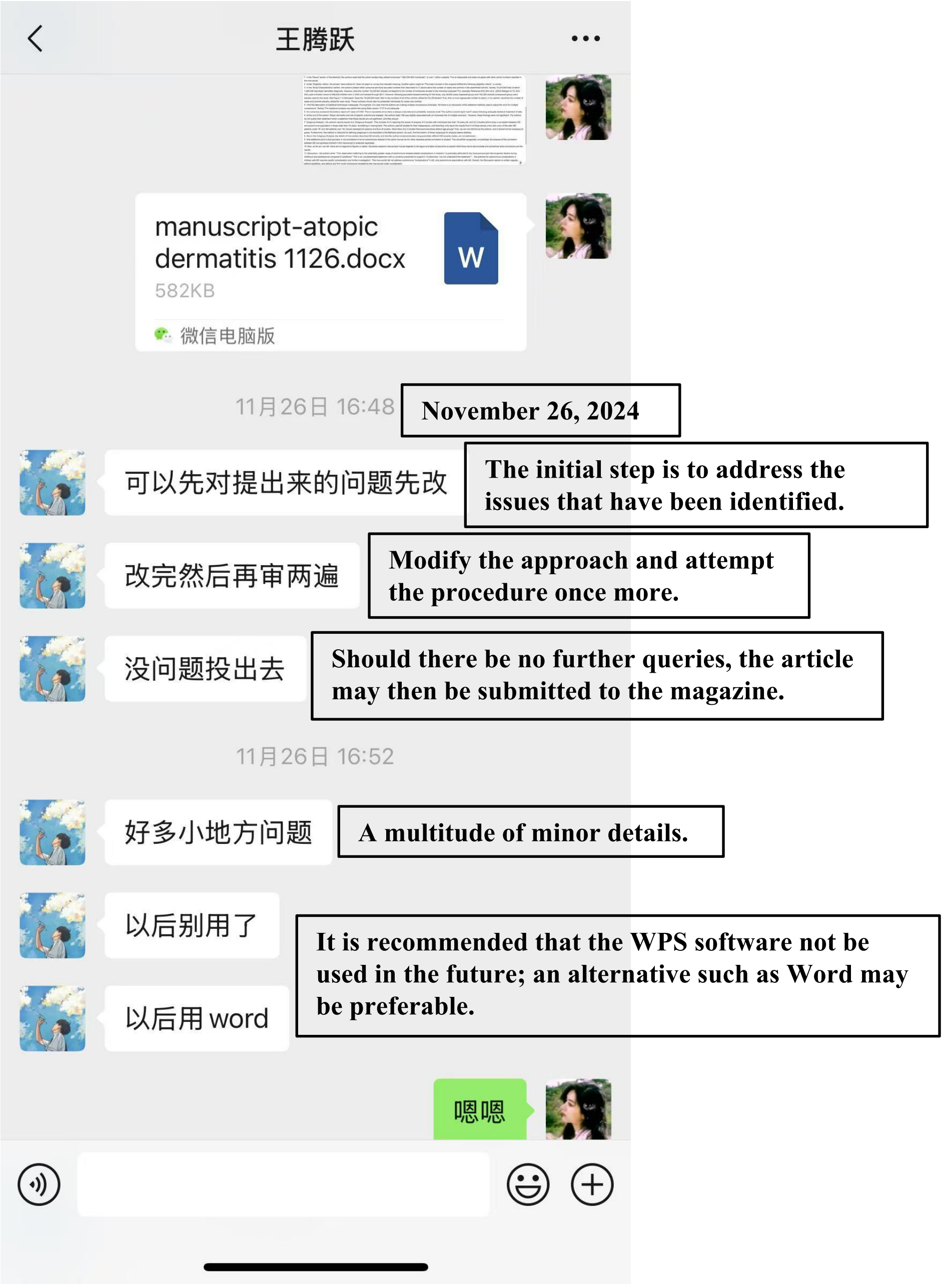

WeChat conversation between Hongli Wang and Tengyue Wang about the research

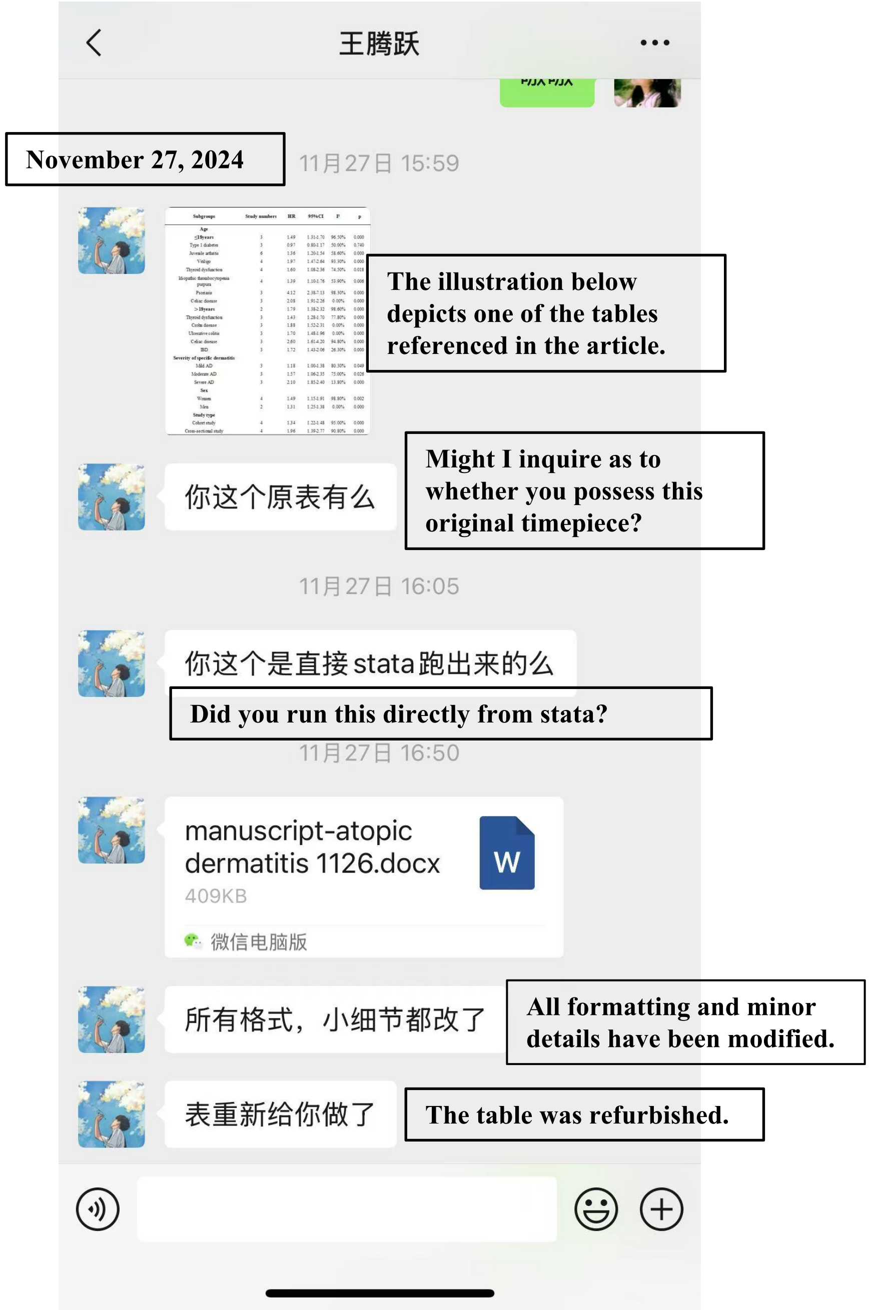

WeChat conversation between Hongli Wang and Lin Huang about the research

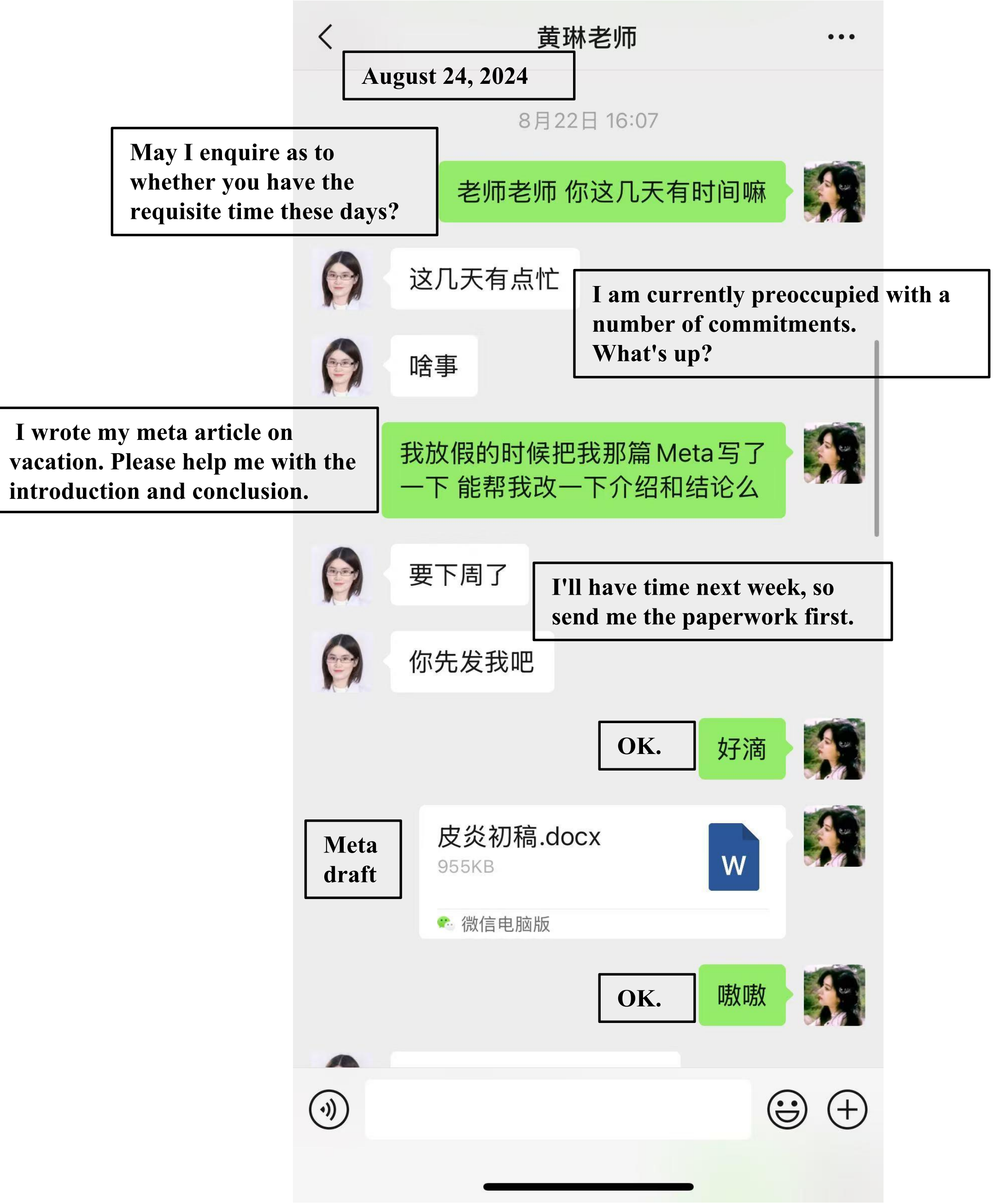

WeChat conversation between Hongli Wang and Lin Huang about the research

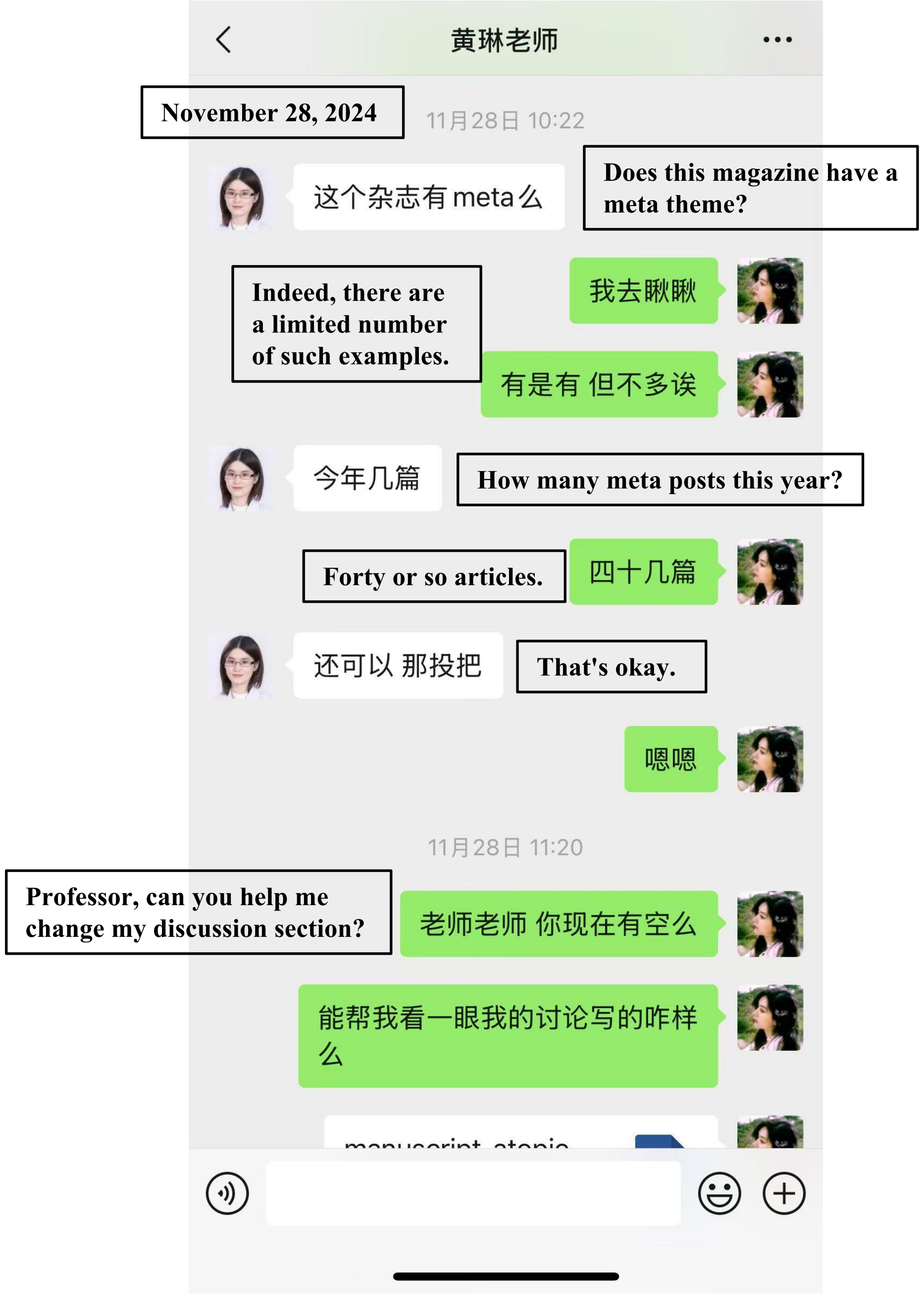

WeChat conversation between Hongli Wang and Mingzhu Wang about the research

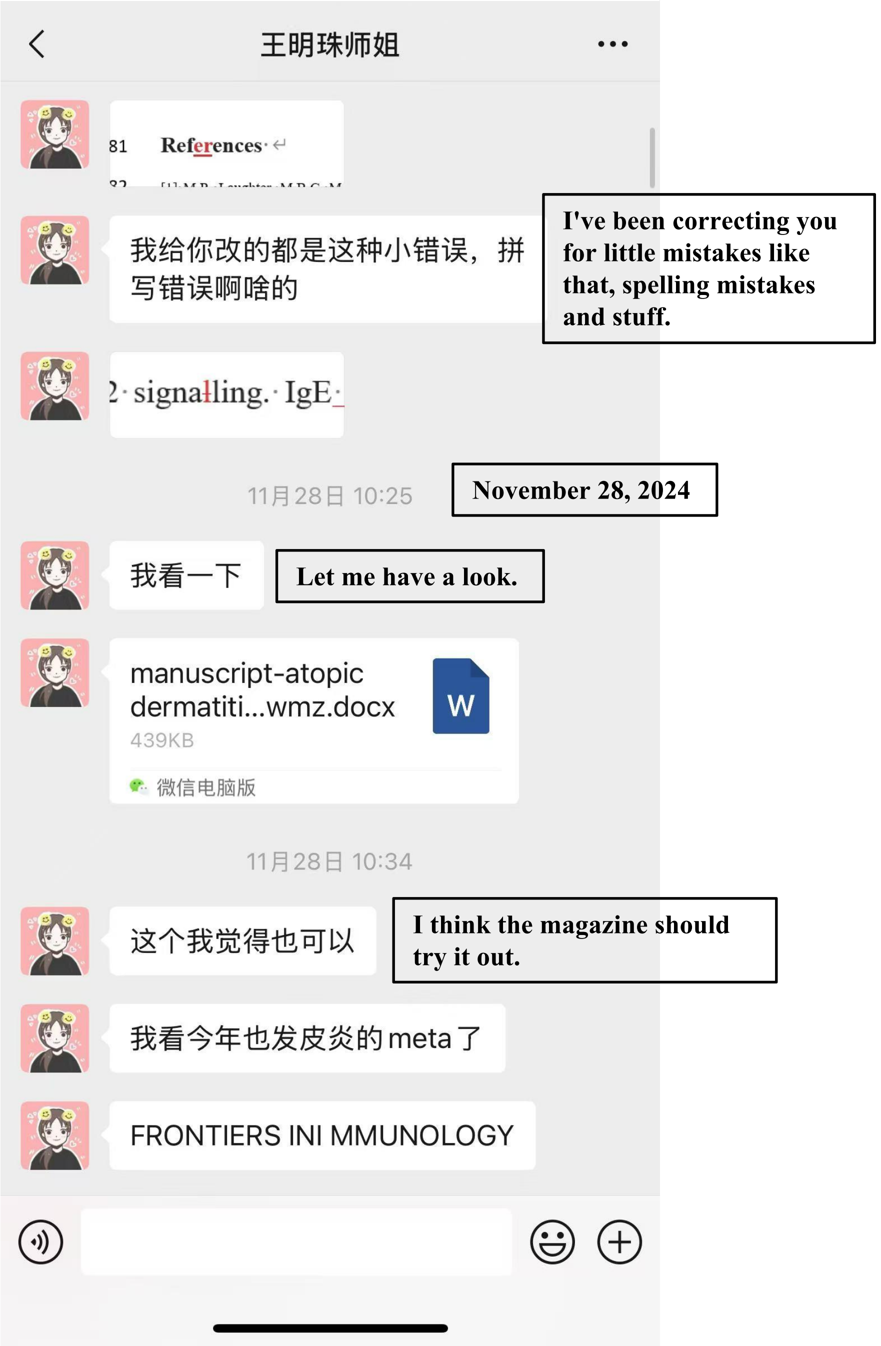

WeChat conversation between Hongli Wang and Mingzhu Wang about the research

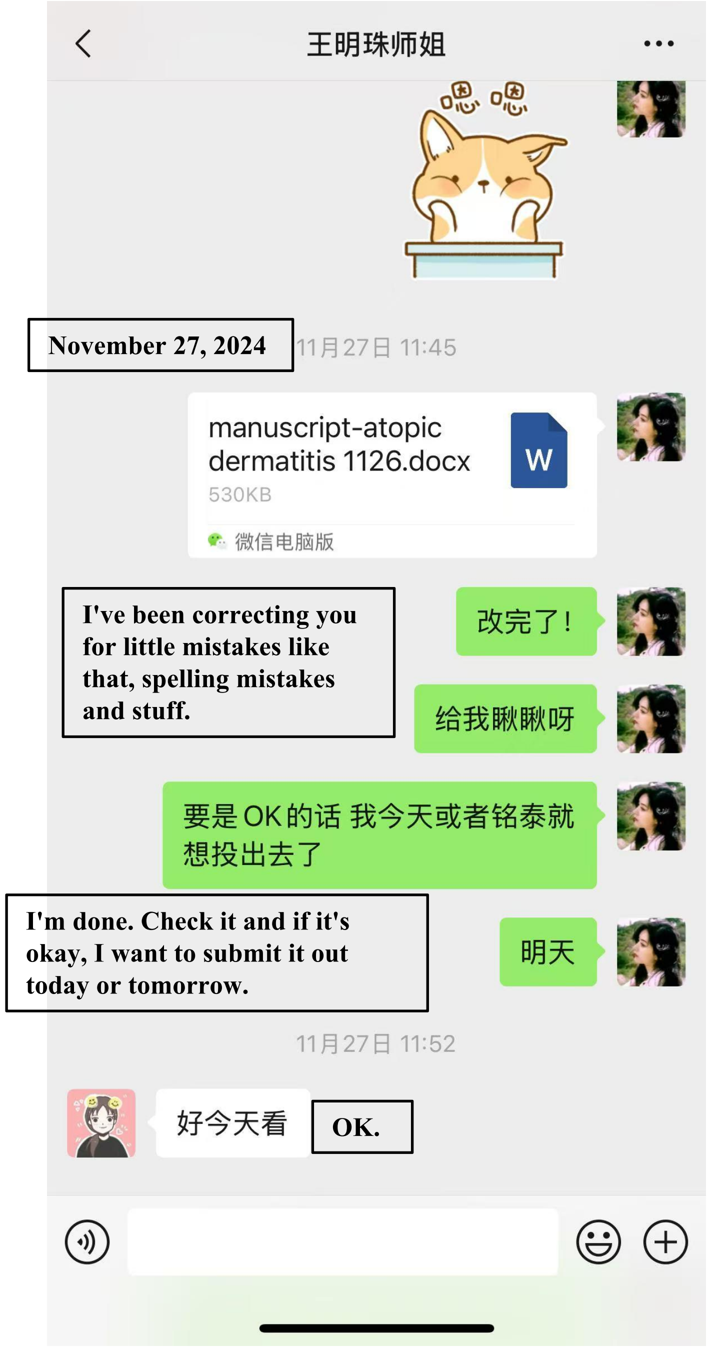

WeChat conversation between Hongli Wang and Wenyu Cai about the research

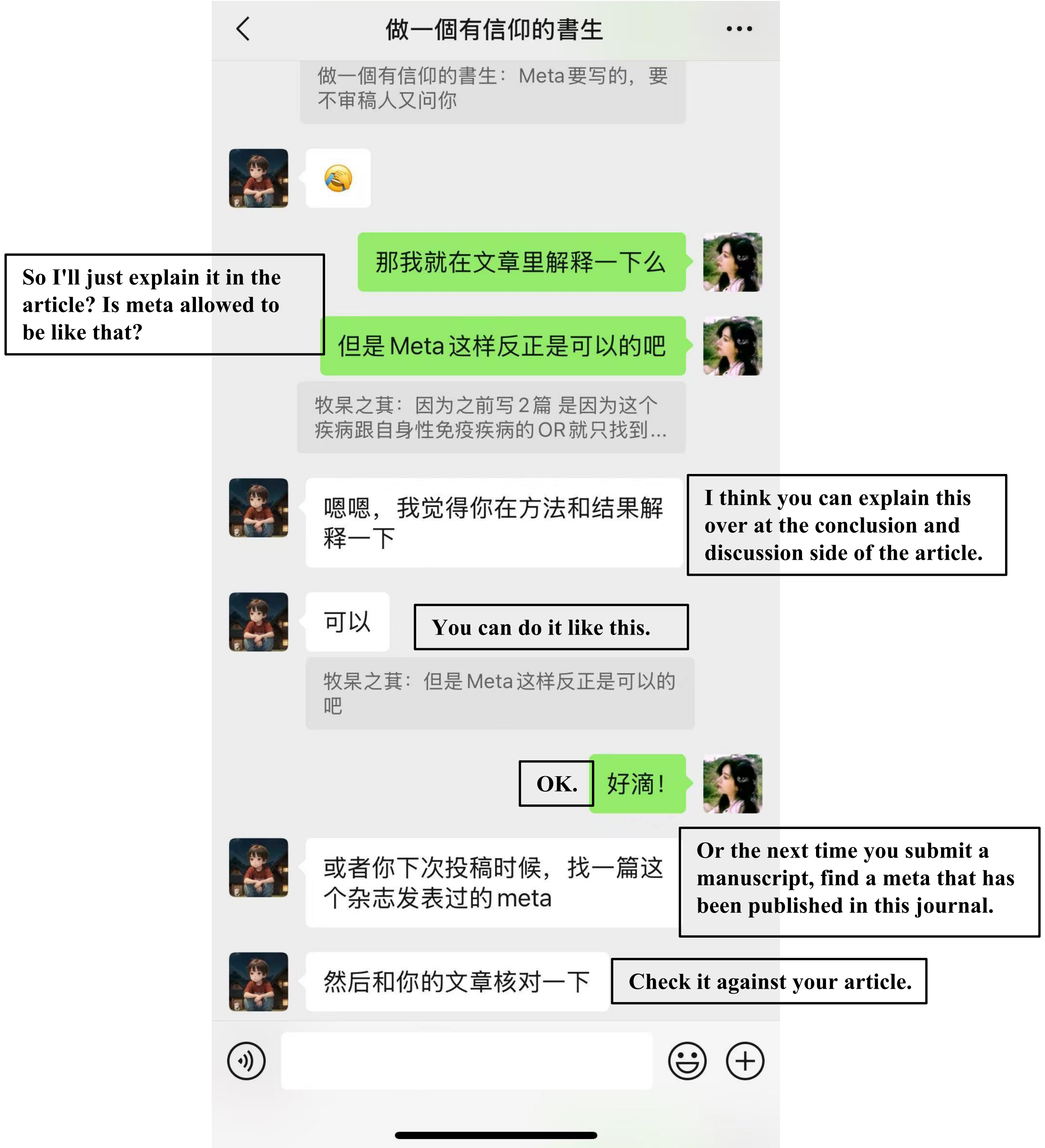

WeChat conversation between Hongli Wang and Wenyu Cai about the research

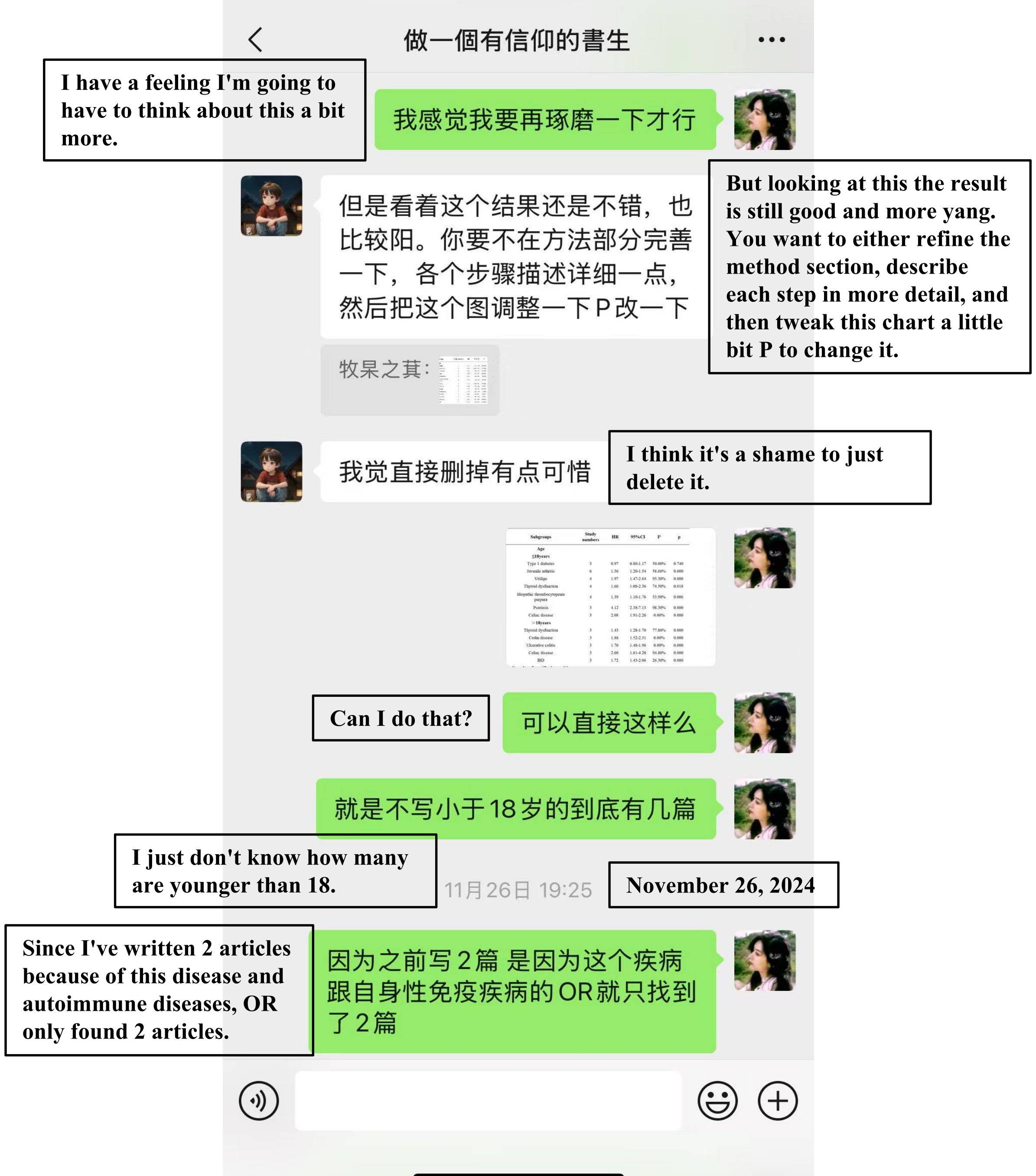

Supplement: Supplementary file 4 [file SupplementaryFile4.pdf]
