# Supplementary material for: Atopic dermatitis and risk of autoimmune diseases: a systematic review and meta-analysis
Source: Front Immunol. 2025 Jun 12;16:1539997. doi: 10.3389/fimmu.2025.1539997 (PMC12198157; doi:10.3389/fimmu.2025.1539997)
Supplement: Supplementary file 5 [file SupplementaryFile5.docx]

**Forest plot demonstrating the association between atopic dermatitis and risk of specific autoimmune diseases**


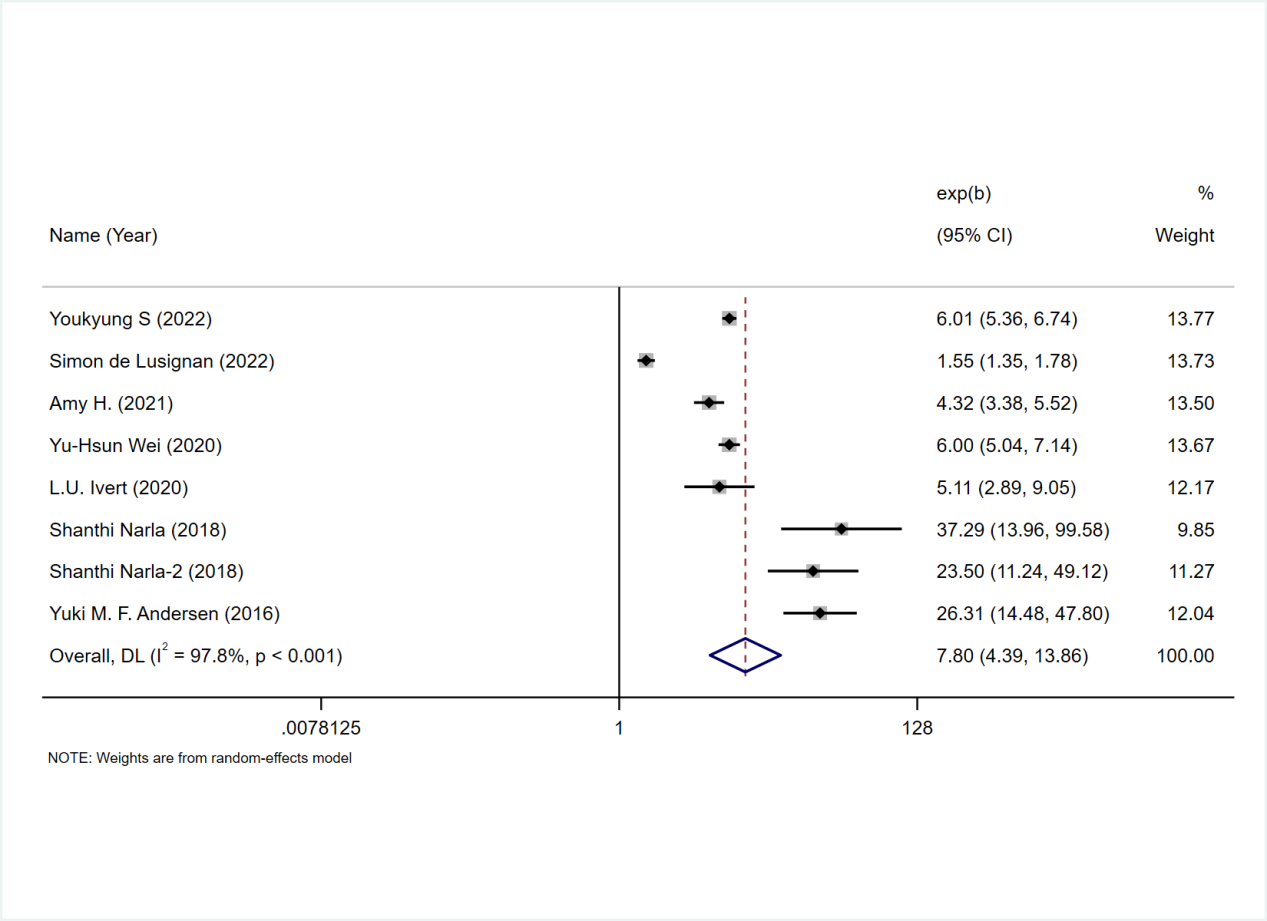


Figure 1. A forest plot illustrated the relationship between atopic dermatitis and alopecia areata risk.


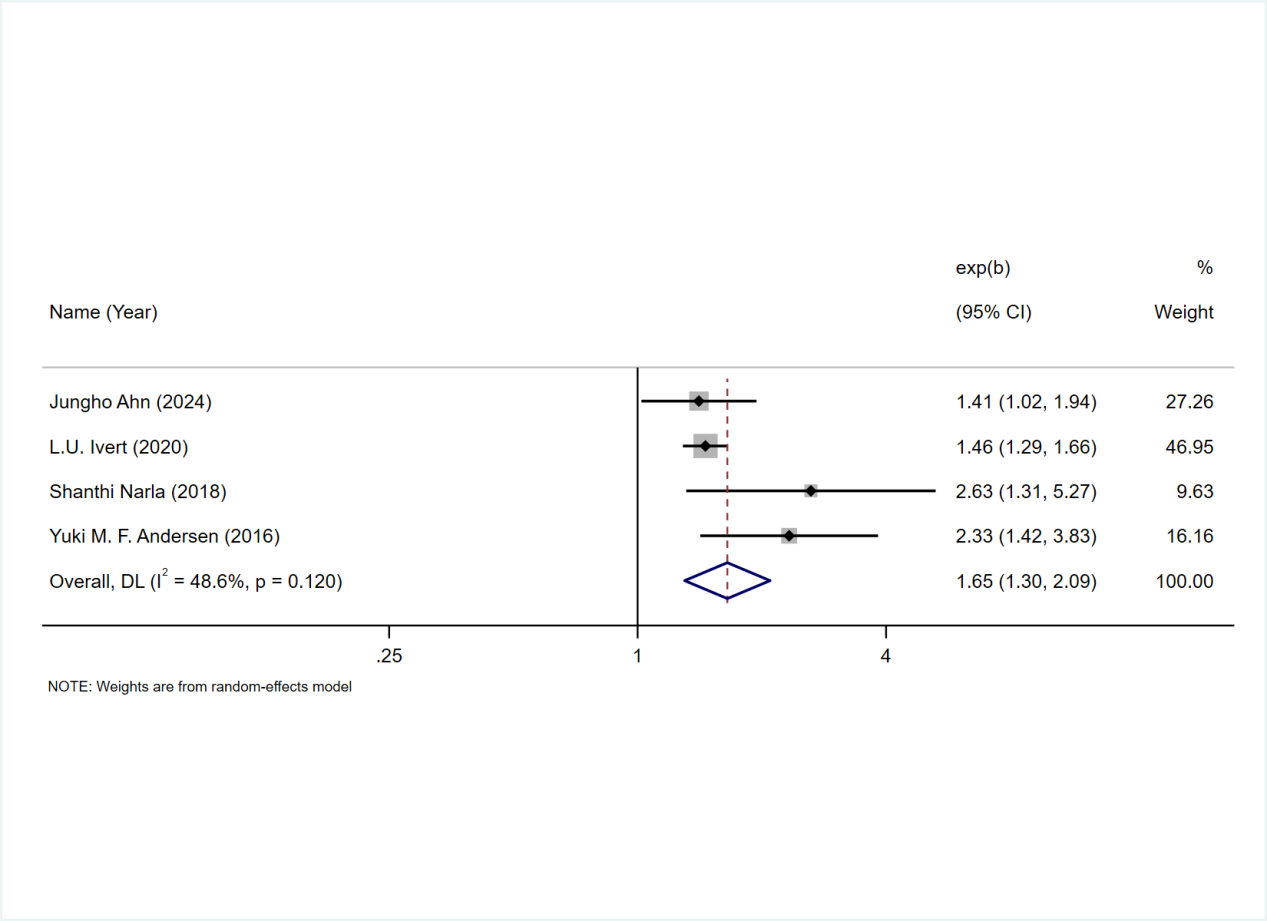


Figure 2. A forest plot illustrated the relationship between atopic dermatitis and ankylosing spondylitis risk.


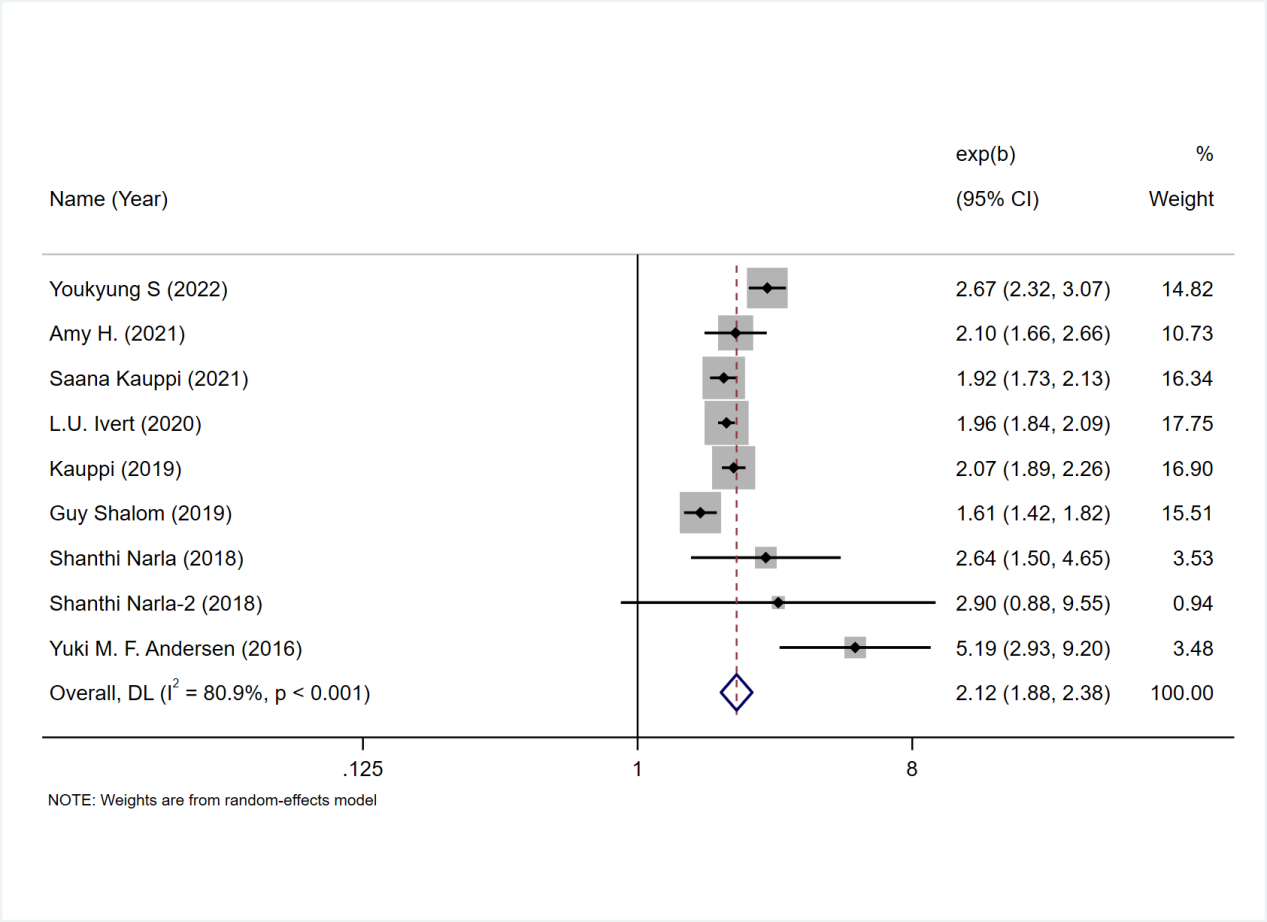


Figure 3. A forest plot illustrated the relationship between atopic dermatitis and celiac disease risk.


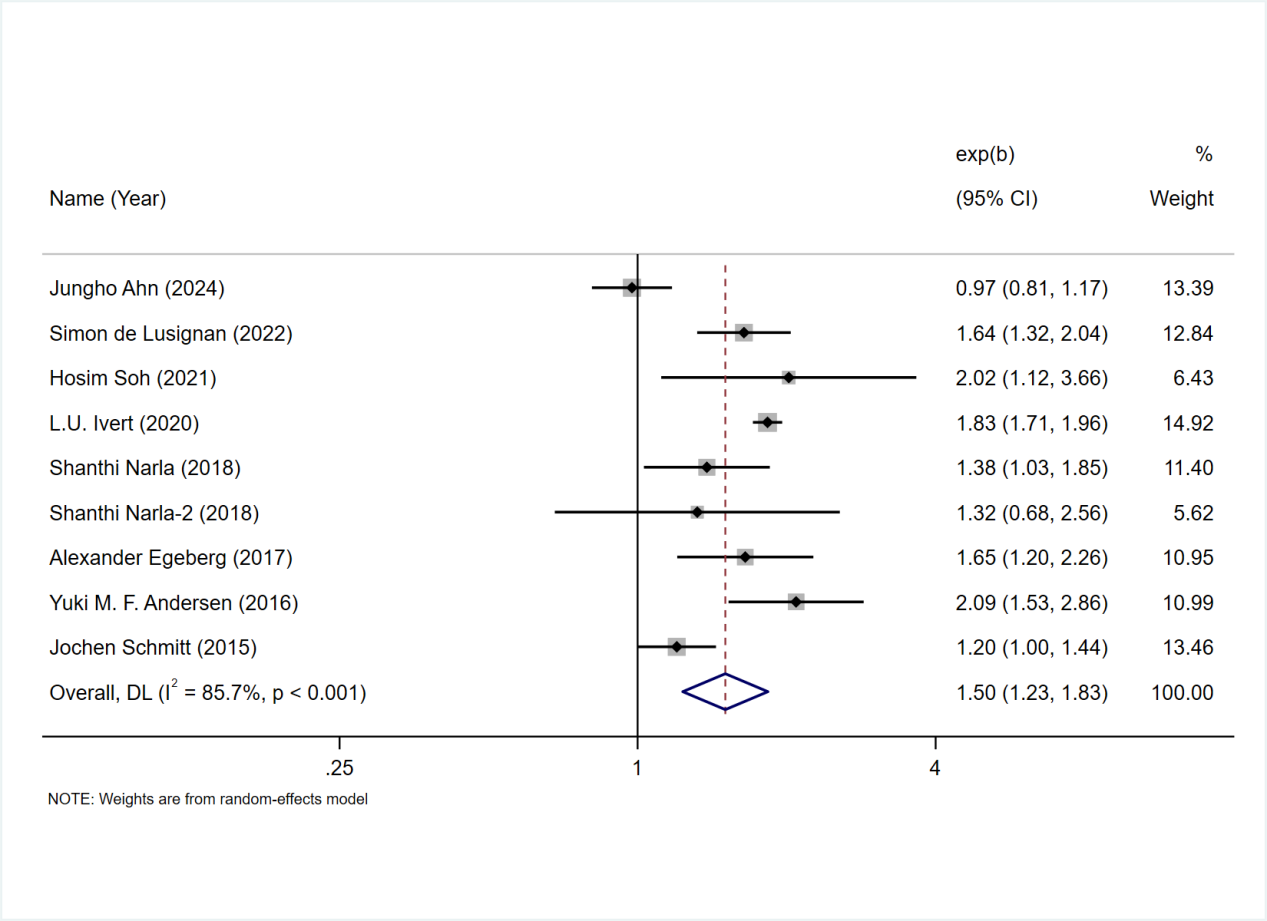


Figure 4. A forest plot illustrated the relationship between atopic dermatitis and Crohn’s disease risk.


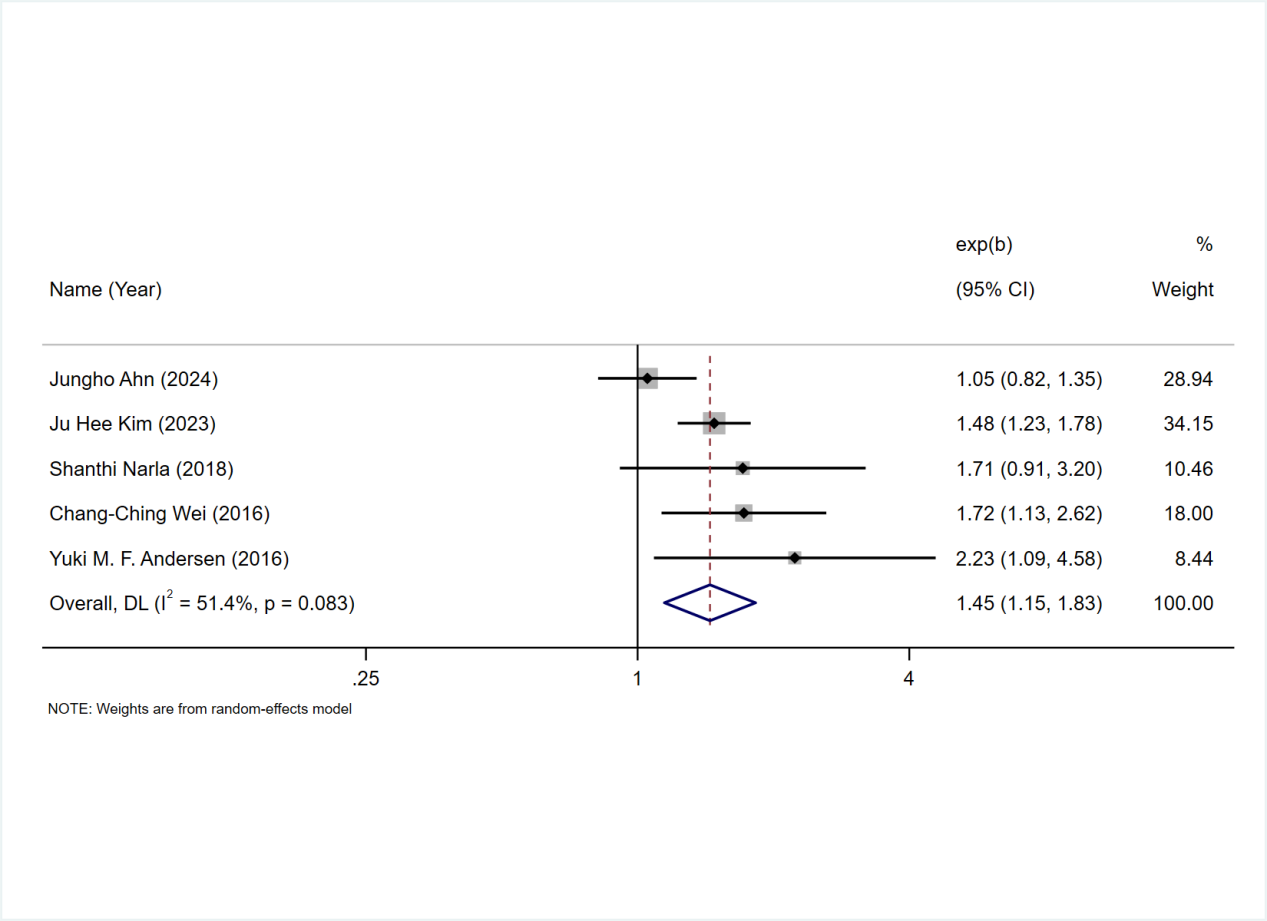


Figure 5. A forest plot illustrated the relationship between atopic dermatitis and idiopathic thrombocytopenia purpura risk.


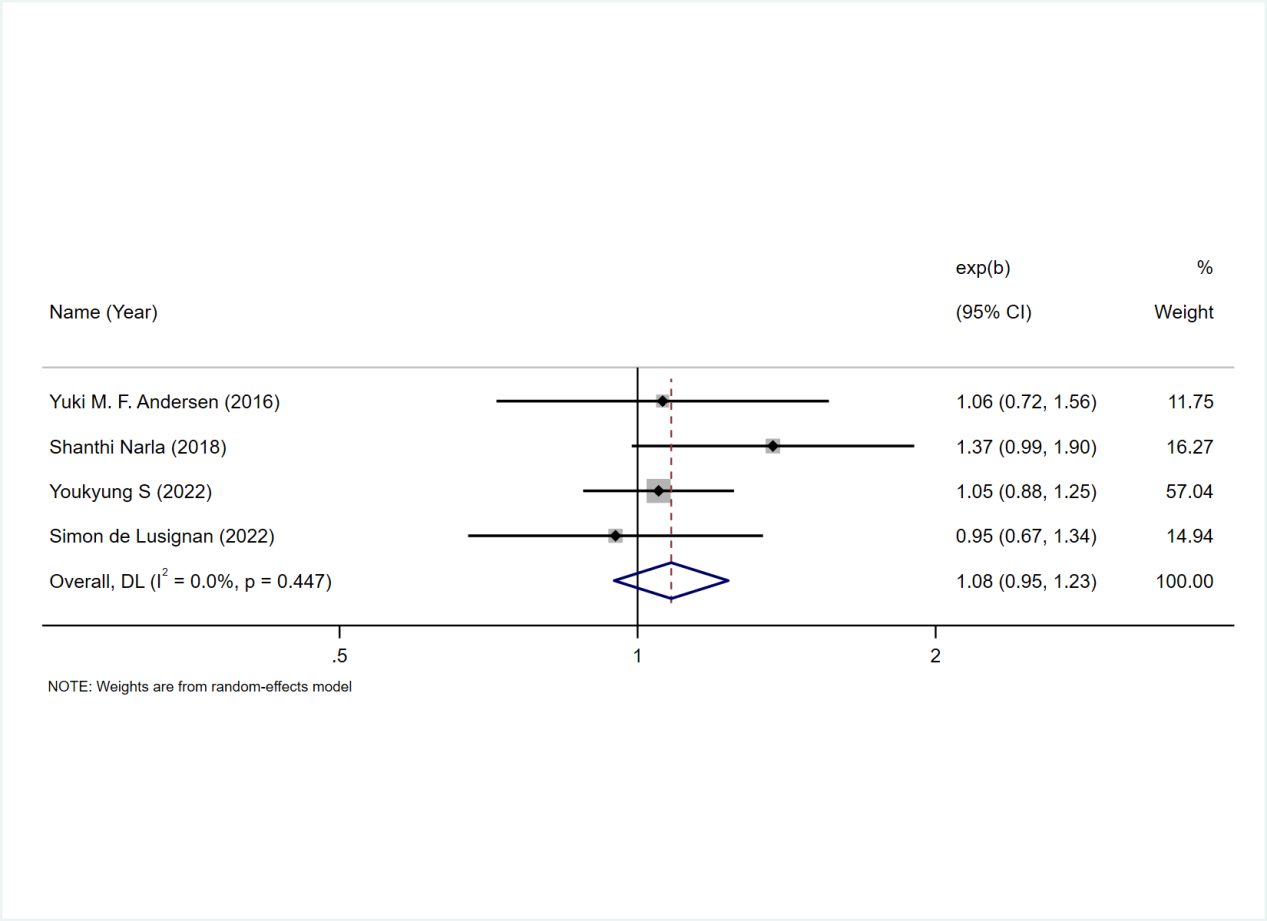


Figure 6. A forest plot illustrated the relationship between atopic dermatitis and multiple sclerosis risk.


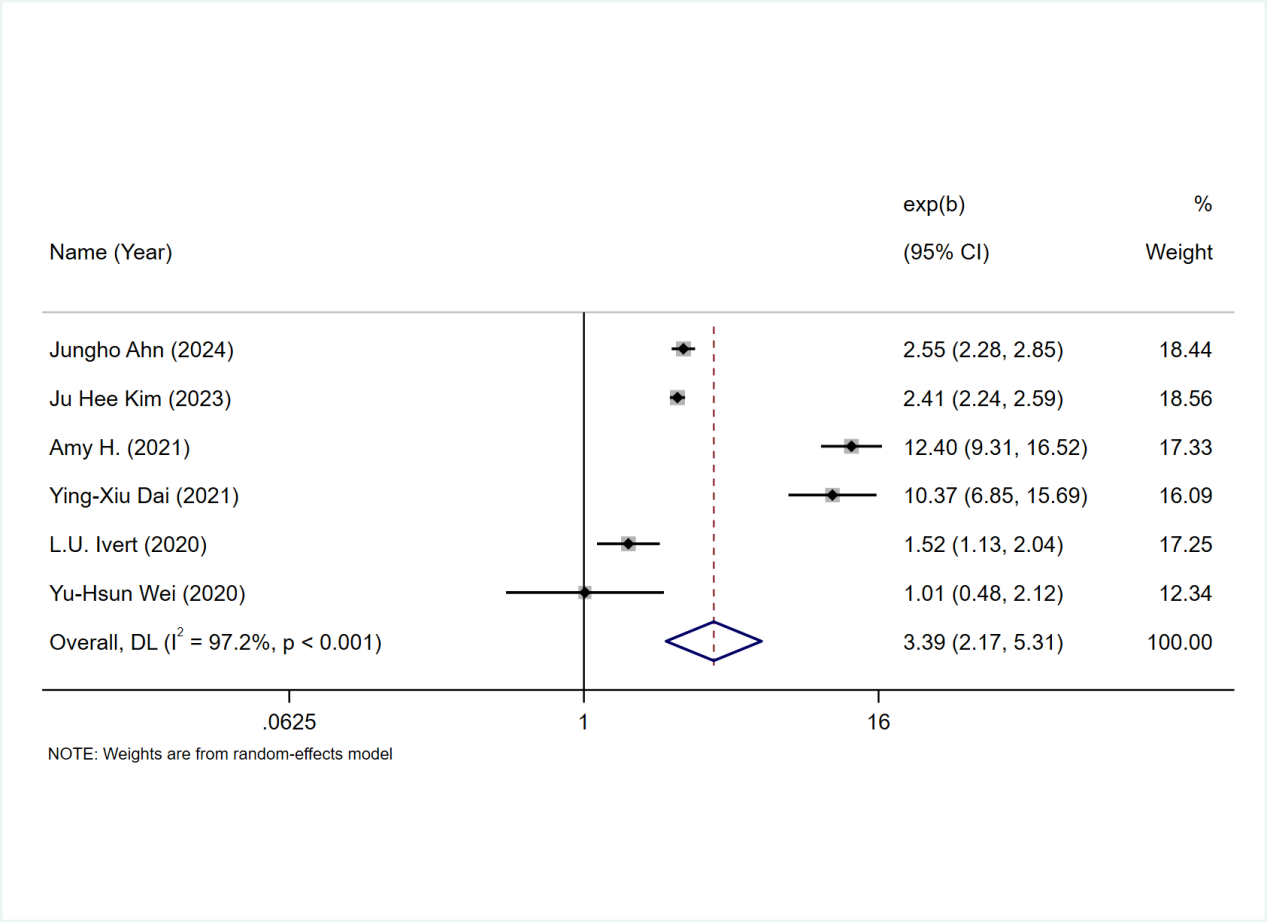


Figure 7. A forest plot illustrated the relationship between atopic dermatitis and psoriasis risk.


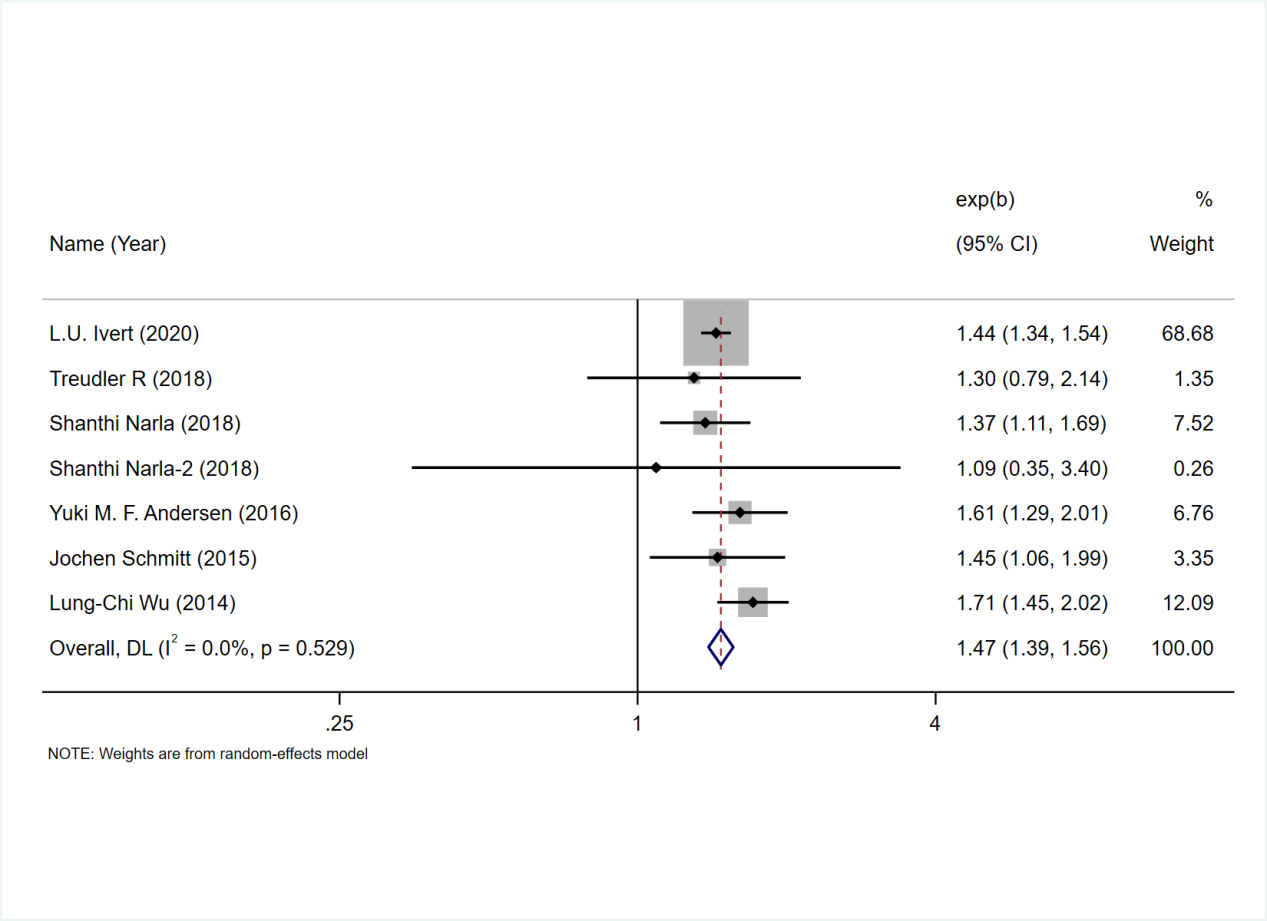


Figure 8. A forest plot illustrated the relationship between atopic dermatitis and rheumatoid arthritis risk.


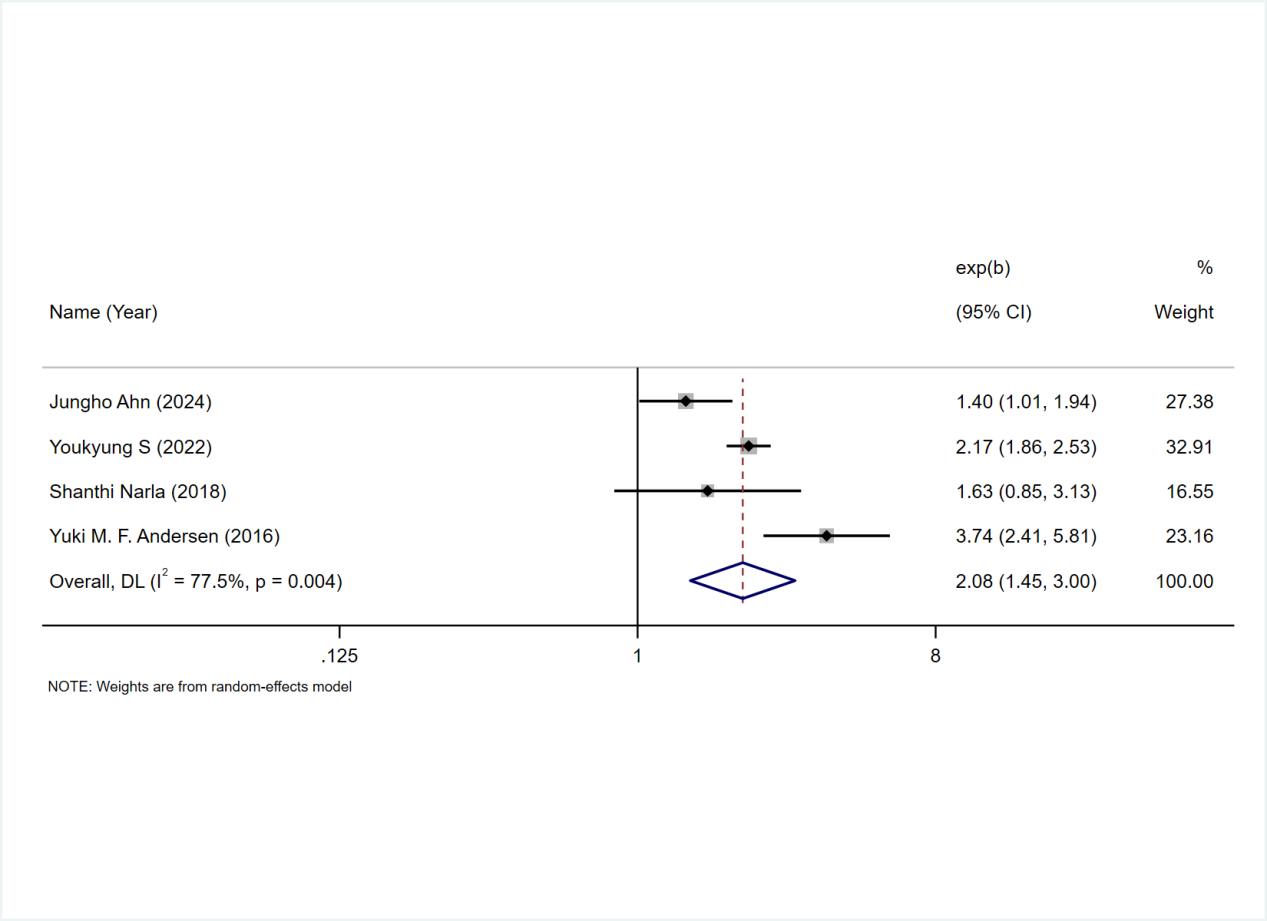


Figure 9. A forest plot illustrated the relationship between atopic dermatitis and Sjögren’s syndrome risk.


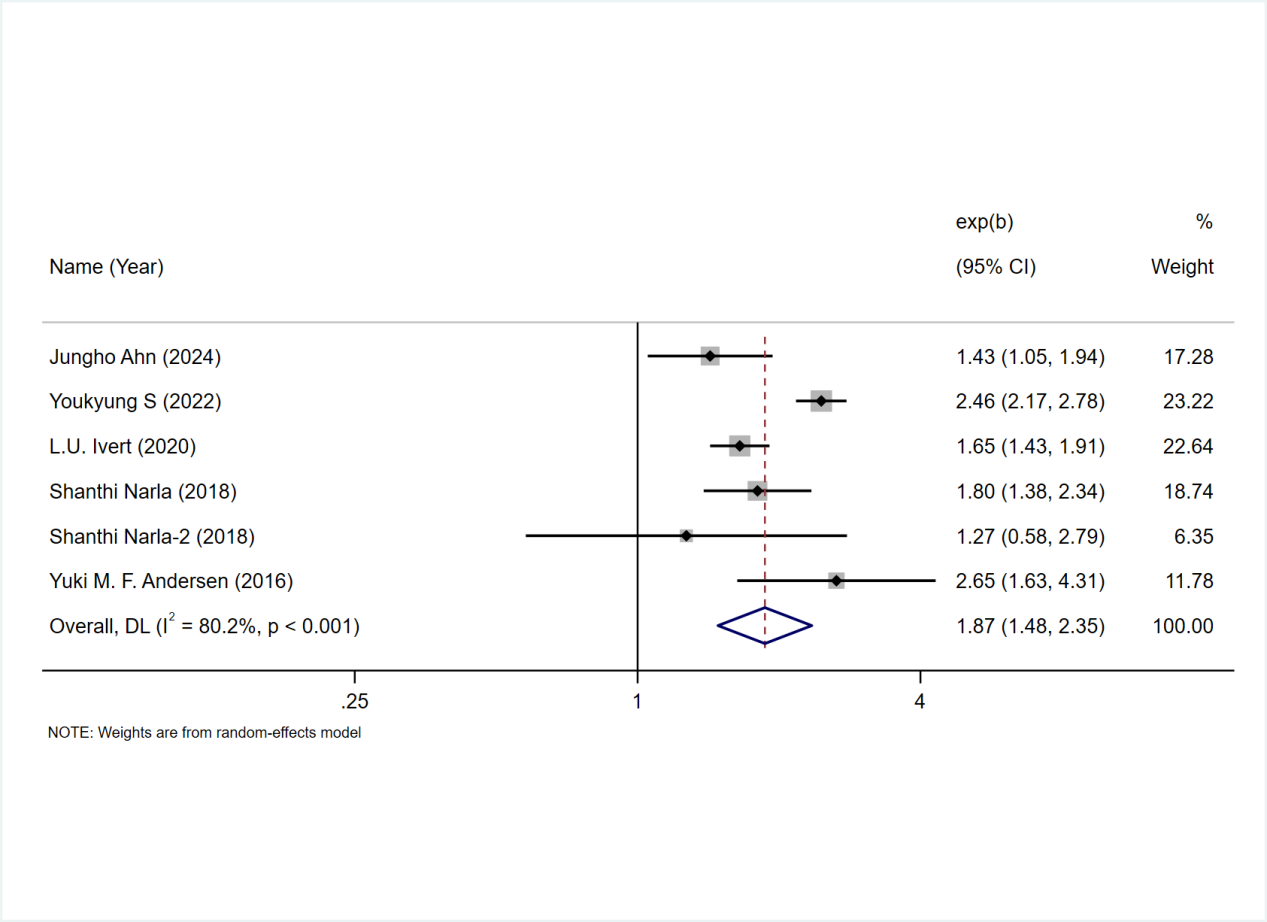


Figure 10. A forest plot illustrated the relationship between atopic dermatitis and systemic lupus erythematosus risk.


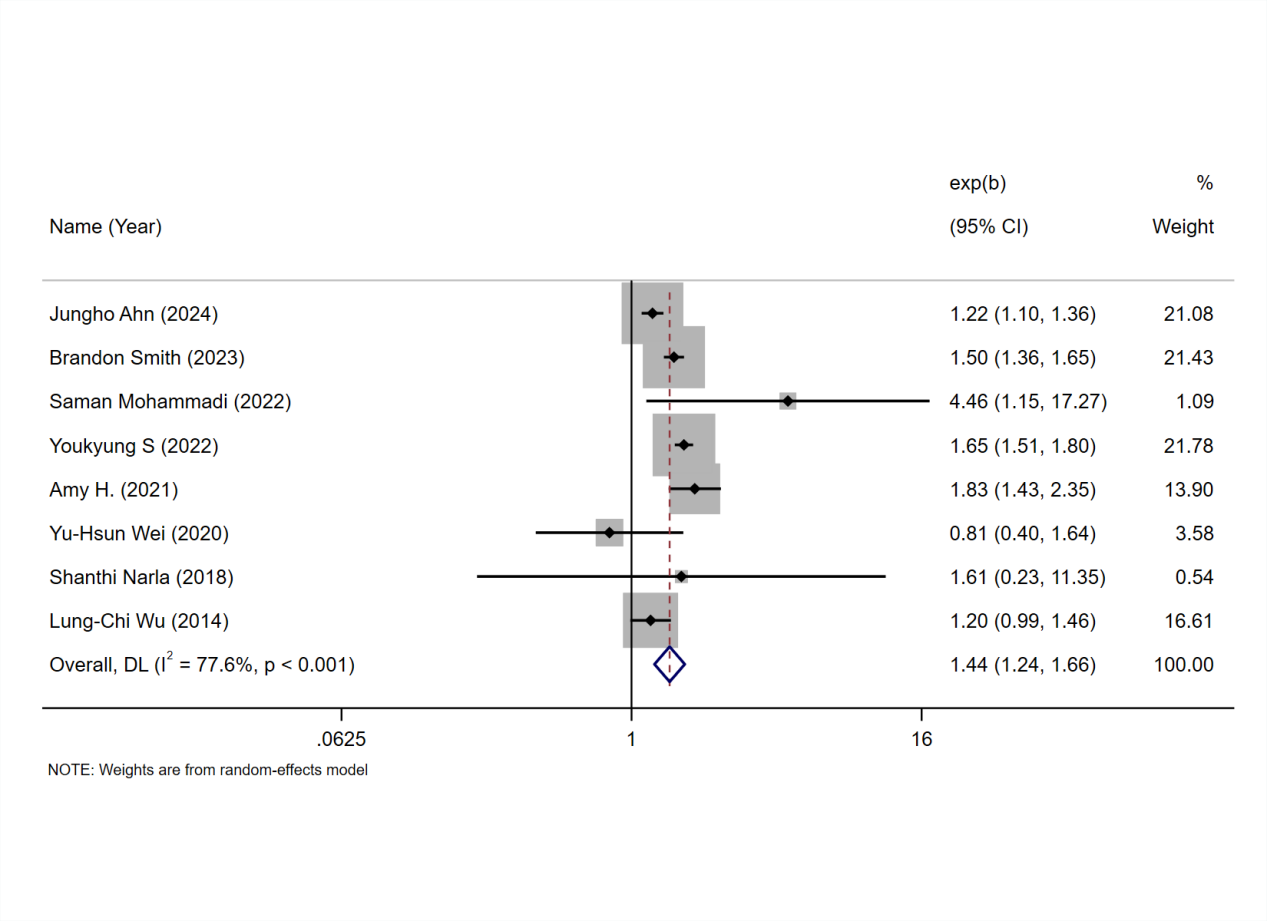


Figure 11. A forest plot illustrated the relationship between atopic dermatitis and thyroid dysfunction risk.


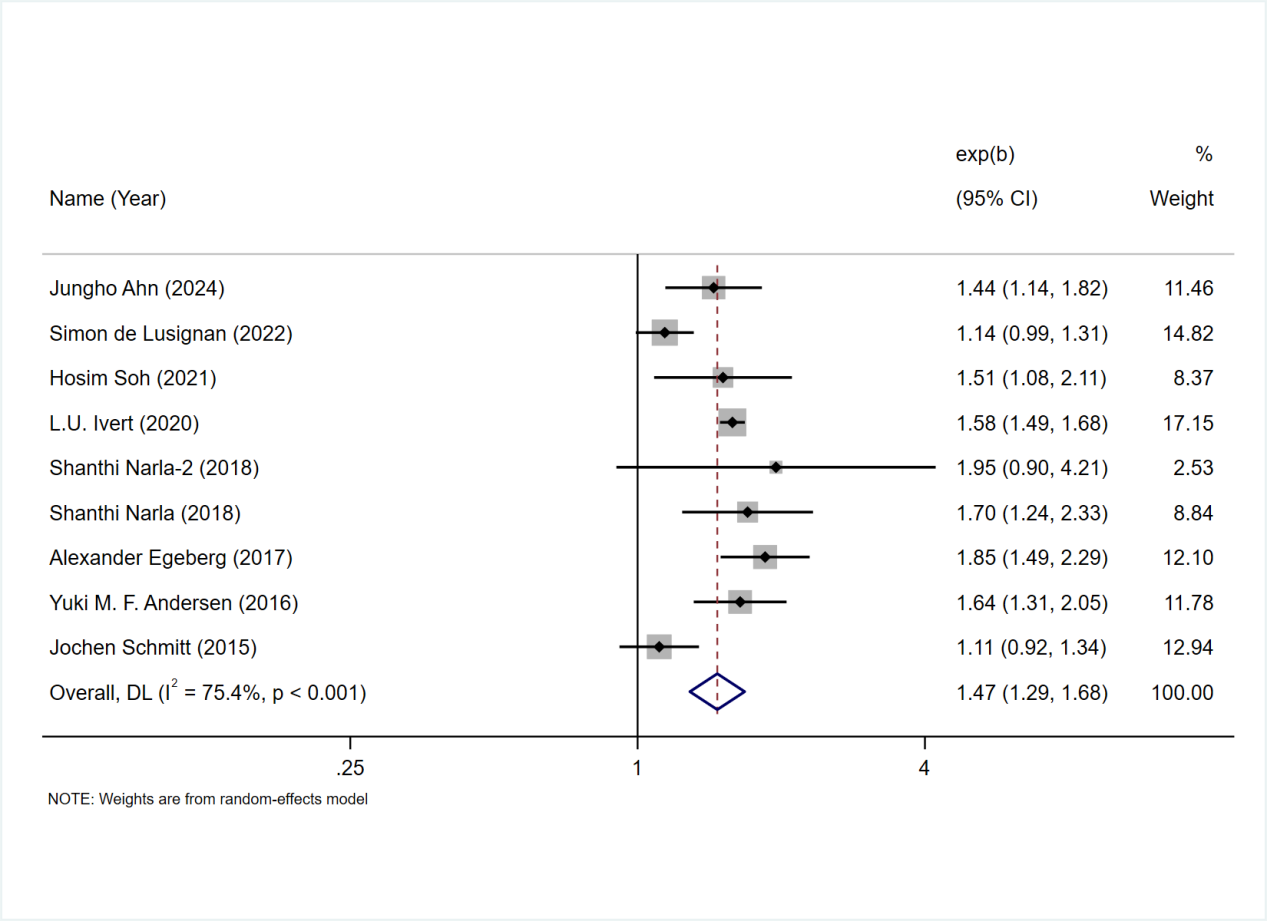


Figure 12. A forest plot illustrated the relationship between atopic dermatitis and ulcerative colitis risk.


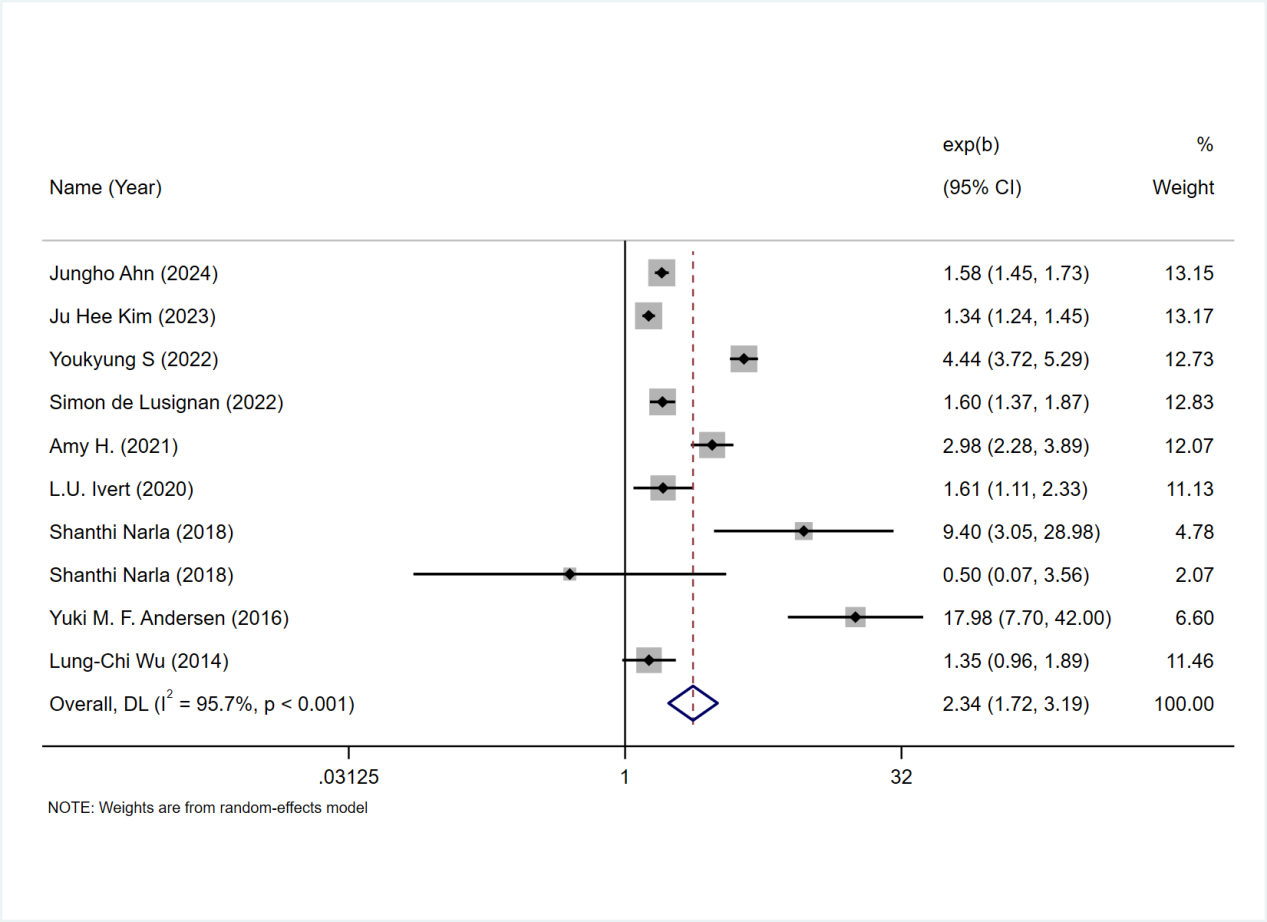


Figure 13. A forest plot illustrated the relationship between atopic dermatitis and vitiligo risk.
