# Supplementary material for: Atopic dermatitis and risk of autoimmune diseases: a systematic review and meta-analysis
Source: Front Immunol. 2025 Jun 12;16:1539997. doi: 10.3389/fimmu.2025.1539997 (PMC12198157; doi:10.3389/fimmu.2025.1539997)
Supplement: Supplementary file 6 [file SupplementaryFile6.docx]

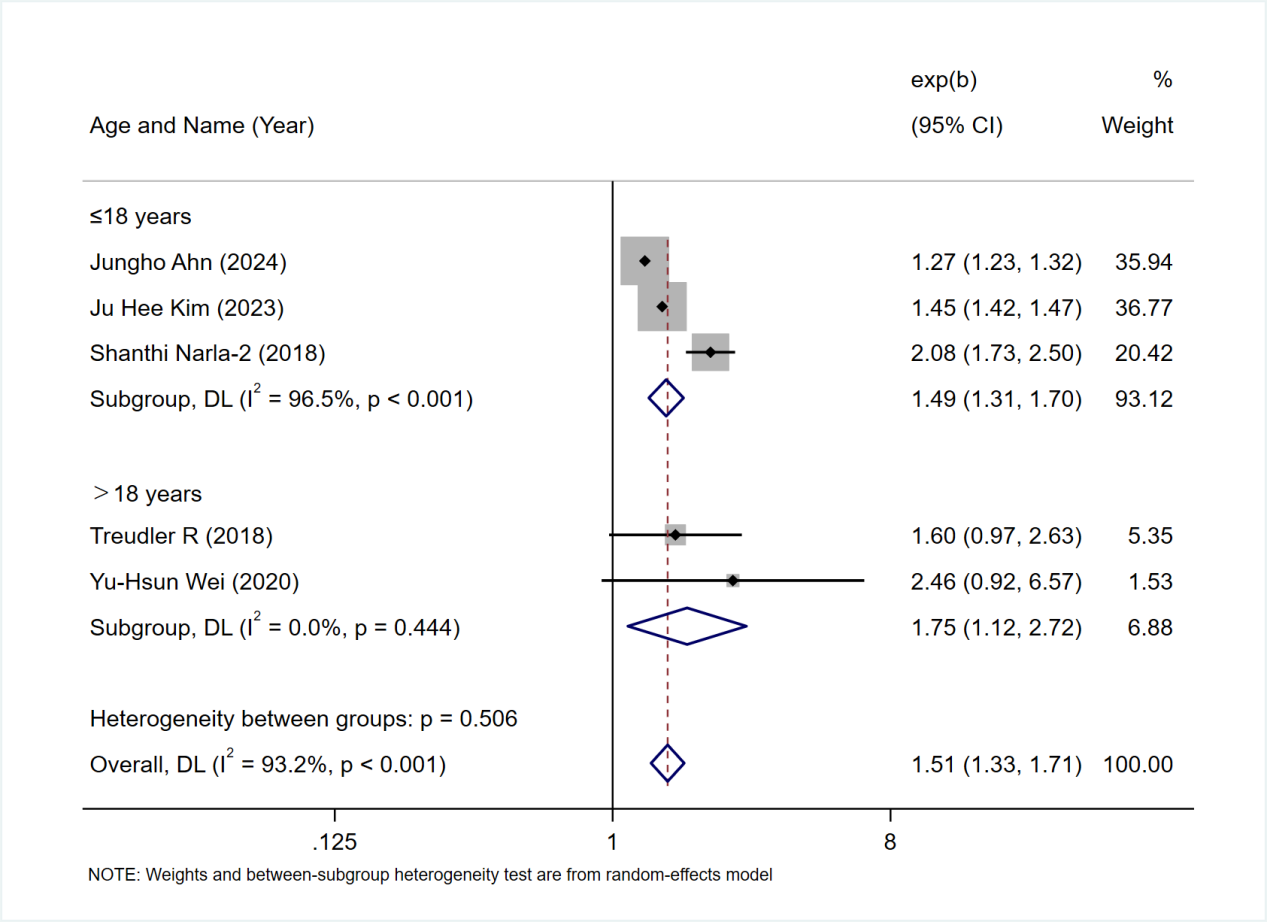


Figure 14. A forest maps of autoimmune disease risk in adults and children with atopic dermatitis.


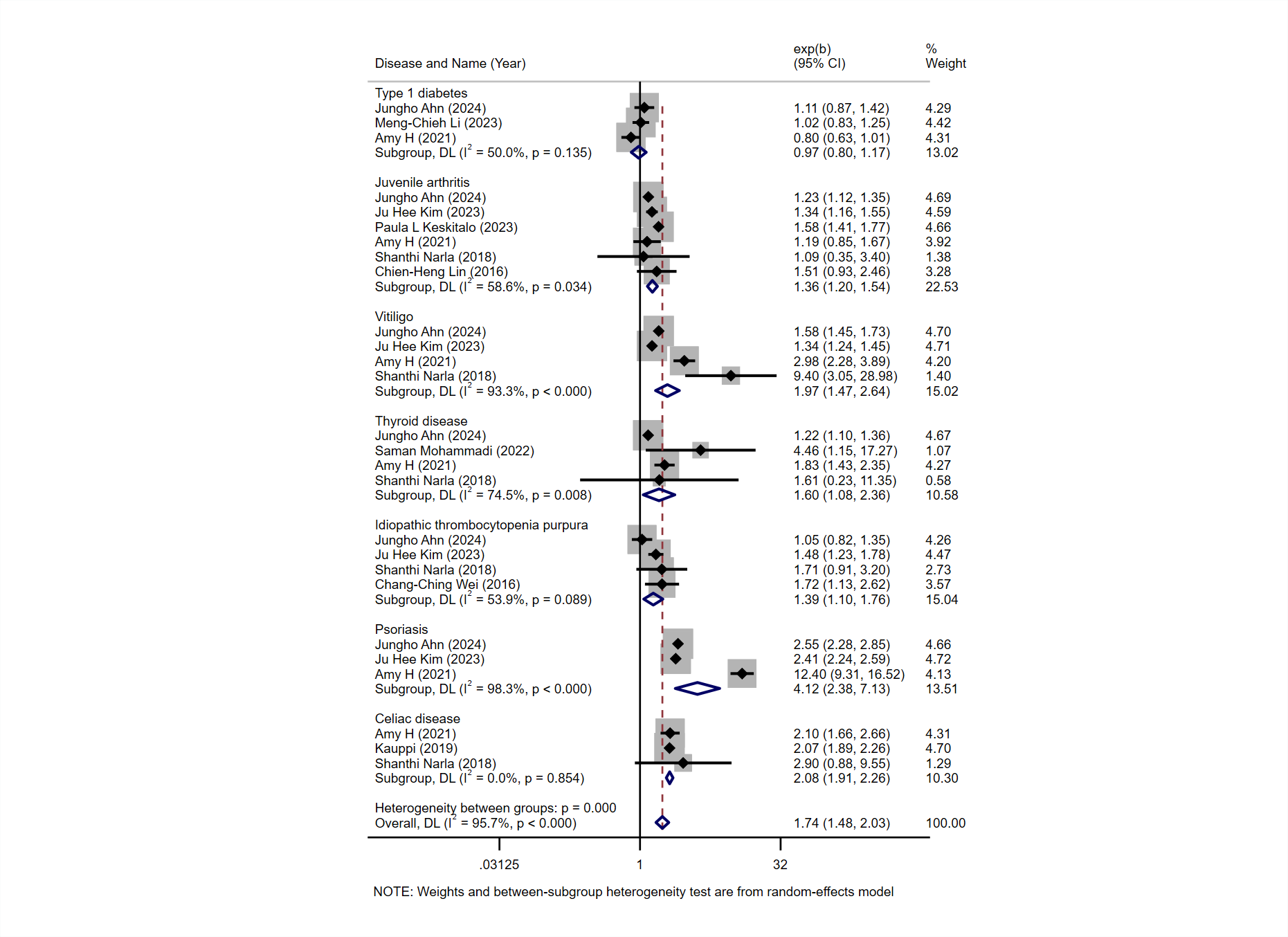


Figure 15. A forest map illustrated the relationship between the age of children with atopic dermatitis and the risk of specific autoimmune diseases.


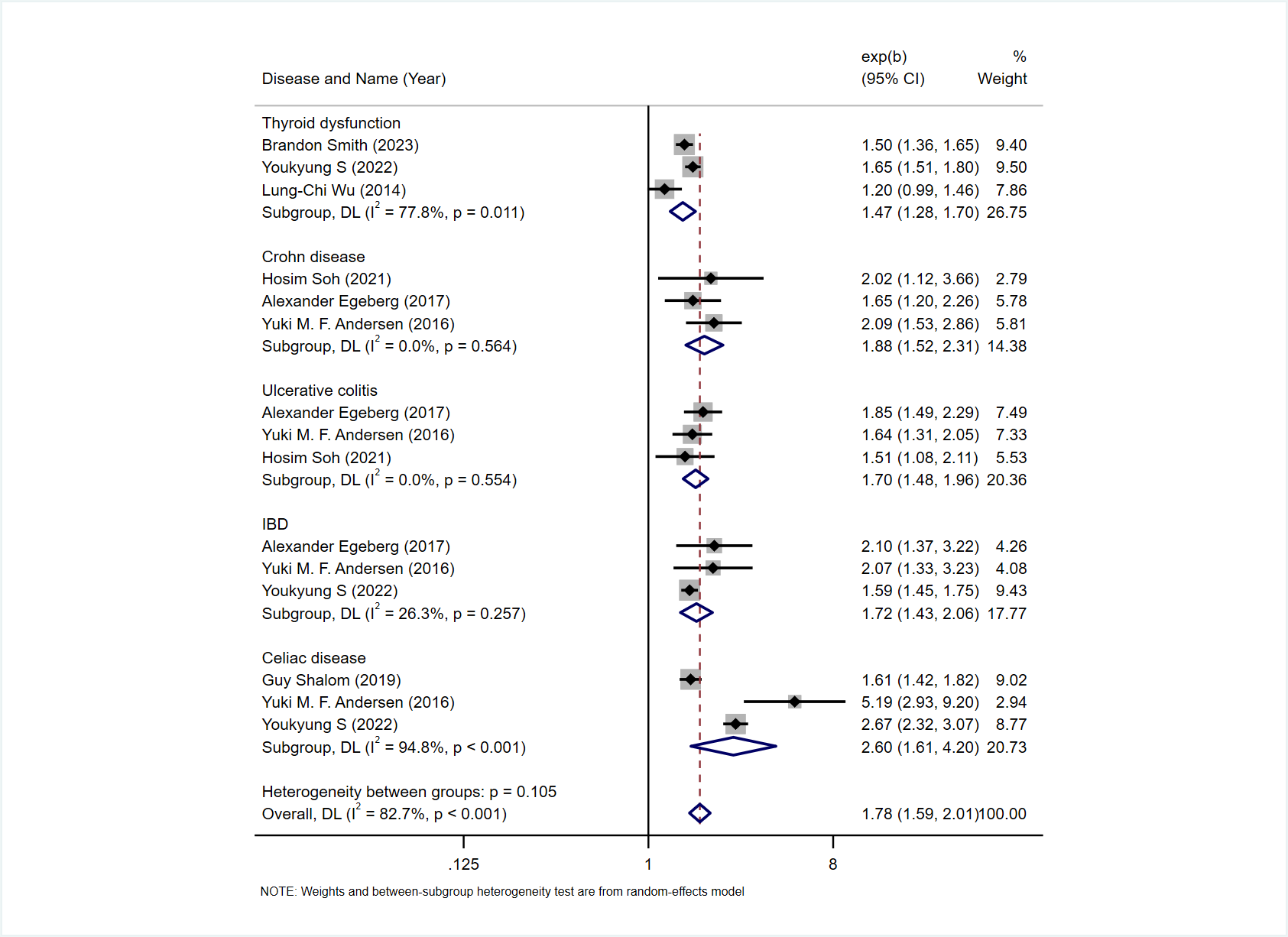


Figure 16. A forest map illustrated the relationship between the age of adults with atopic dermatitis and the risk of specific autoimmune diseases.


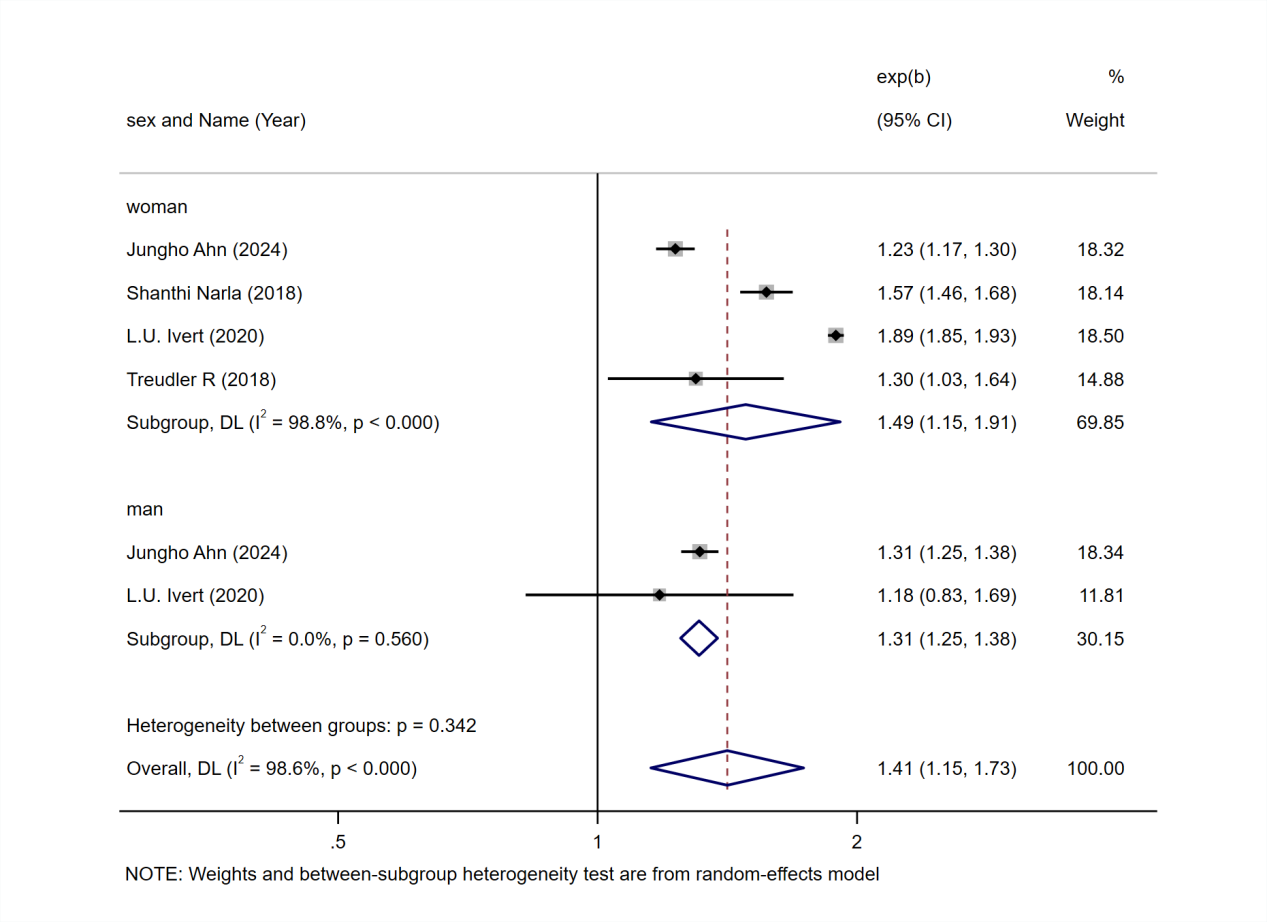


Figure 17. A forest map illustrated the relationship between the gender of people with atopic dermatitis and the risk of specific autoimmune diseases.


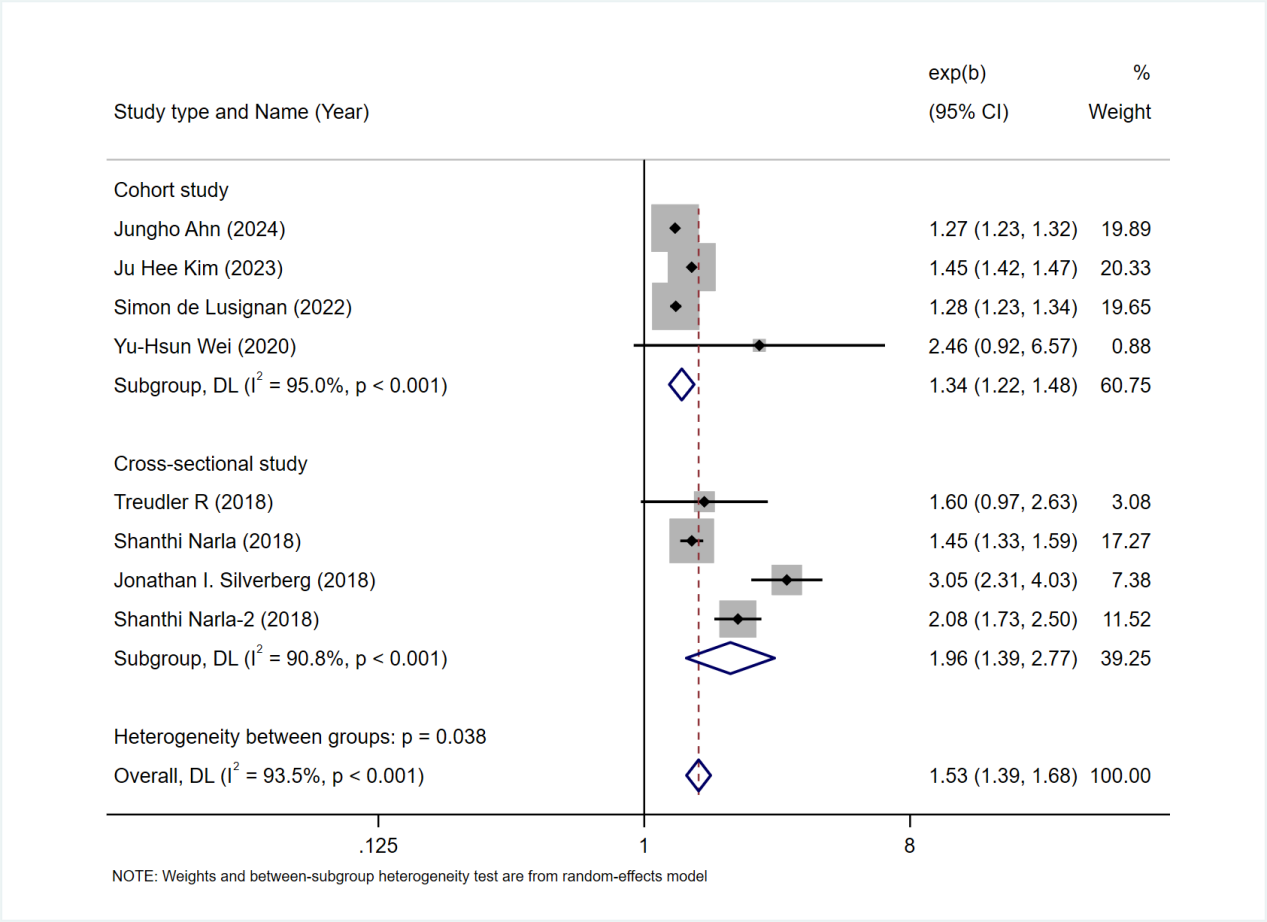


Figure 18. A forest map illustrated the relationship between study type and the risk of specific autoimmune diseases.


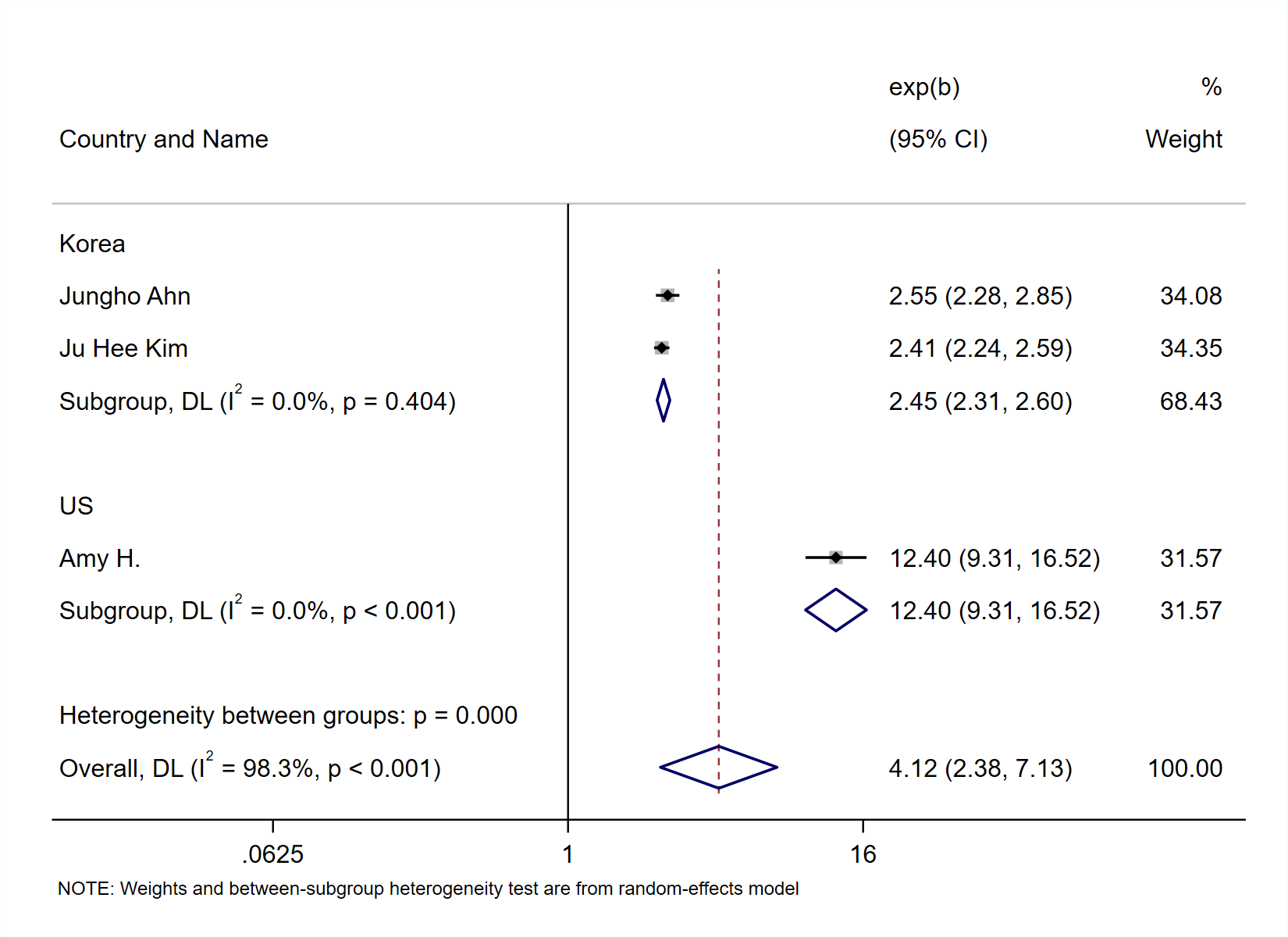


Figure 19. Forest plots depicting subgroup analyses between atopic dermatitis patients aged ≤18 years and the risk of specific autoimmune diseases.
